# The Protective Effect of the Supplementation with an Extract from *Aronia melanocarpa* L. Berries against Cadmium-Induced Changes of Chosen Biomarkers of Neurotoxicity in the Brain—A Study in a Rat Model of Current Lifetime Human Exposure to This Toxic Heavy Metal

**DOI:** 10.3390/ijms252010887

**Published:** 2024-10-10

**Authors:** Agnieszka Ruczaj, Joanna Rogalska, Małgorzata Gałażyn-Sidorczuk, Małgorzata M. Brzóska

**Affiliations:** Department of Toxicology, Medical University of Bialystok, Adama Mickiewicza 2C Street, 15-222 Bialystok, Poland; agnieszka.ruczaj@sd.umb.edu.pl (A.R.); joanna.rogalska@umb.edu.pl (J.R.); malgorzata.galazyn-sidorczuk@umb.edu.pl (M.G.-S.)

**Keywords:** acetylcholinesterase (AChE), adenosine triphosphatase (ATPase), *Aronia melanocarpa* L. berries extract (AE), calmodulin (CAM), markers of neurotoxicity, metalloproteinases and their tissue inhibitors (MMPs and TIMPs), nervous tissue, nitric oxide synthase 1, polyphenolic compounds, protection from cadmium neurotoxicity

## Abstract

Since even low-level environmental exposure to cadmium (Cd) can lead to numerous unfavourable health outcomes, including damage to the nervous system, it is important to recognize the risk of health damage by this xenobiotic, the mechanisms of its toxic influence, and to find an effective protective strategy. This study aimed to evaluate, in a female Wistar rat model of current human environmental exposure to Cd (1 and 5 mg/kg of diet for 3–24 months), if the low-to-moderate treatment with this element can harm the brain and whether the supplementation with a 0.1% *Aronia melanocarpa* L. (Michx.) Elliott berries (chokeberries) extract (AE) can protect against this effect. The exposure to Cd modified the values of various biomarkers of neurotoxicity, including enzymes (acetylcholinesterase (AChE), sodium-potassium adenosine triphosphatase (Na^+^/K^+^-ATPase), phospholipase A2 (PLA2), and nitric oxide synthase 1 (NOS1)) and non-enzymatic proteins (calmodulin (CAM), nuclear factor erythroid 2-related factor 2 (Nrf2), and Kelch-like ECH-associated protein 1 (KEAP1)) crucial for the functioning of the nervous system, as well as the concentrations of calcium (Ca) and magnesium (Mg) and some metalloproteinases (MMPs) and their tissue inhibitors (TIMPs) in the brain tissue. The co-administration of AE, partially or entirely, protected from most of the Cd-induced changes alleviating its neurotoxic influence. In conclusion, even low-level chronic exposure to Cd may adversely affect the nervous system, whereas the supplementation with *A. melanocarpa* berries products during the treatment seems a protective strategy.

## 1. Introduction

Numerous natural products rich in biologically active substances are known for their effectiveness in preventing various health disorders, including nervous system diseases, and supporting their treatment [1,2]. As the morbidity of nervous system diseases, including neurodegenerative ones, is growing [3,4], it is essential to understand better their etiopathogenetic factors, especially the possible involvement of environmental pollutants. One such substance is cadmium (Cd)—a toxic heavy metal to which exposure is inevitable in developed countries [5,6,7]. Unfavourable health outcomes, including participation in the development of Alzheimer’s disease and cognitive disorders, were reported at current environmental exposure to this xenobiotic [4,8,9]. Thus, it is important to recognize the impact of low-to-moderate intoxication with this harmful element on the nervous system, as well as to find potential protective agents.

Although the harmful effect of Cd on the nervous system has been known for a long time [7,9], it is not entirely clear how this xenobiotic exerts its neurotoxic effects, especially at a low exposure. An important mechanism seems to be the induction of oxidative stress in the brain via dysregulation of the balance between oxidants and antioxidants [10,11,12,13,14,15,16,17,18,19,20,21,22,23,24,25]. Cd can influence factors that control the antioxidative status of the brain, such as, among other things, the nuclear factor erythroid 2-related factor 2 (Nrf2) and Kelch-like ECH-associated protein 1 (KEAP1), which negatively regulates Nrf2 [15,22,23,26]. Nrf2 controls the redox balance via activating non-enzymatic (including binding to the antioxidant response element—ARE—sequence) and enzymatic antioxidants, and changes in its activity influence the susceptibility to the development of oxidative stress in different organs under exposure to Cd [22,23,26]. Moreover, it has been reported to increase the concentration of nitric oxide synthase 1 (NOS1; neuronal NOS), a sensitive marker of oxidative damage to the nervous tissue [16]. Because Cd can induce oxidative stress even at low concentrations in the nervous tissue [20] and the destruction of the oxidative–antioxidative balance in the nervous cells has numerous negative outcomes [27], a better understanding of the mechanism of the pro-oxidative effect of this harmful metal, as well as the identification of the processes disrupted by the induction of oxidative stress, are necessary. Oxidative stress in the brain tissue can lead to an increase in the activity of matrix metalloproteinases (MMPs) [28] and an elevation of the concentration of calcium ions (Ca^2+^) which are second messengers in the nervous system but in excessive amounts are cytotoxic [29]. The co-occurrence of the disrupted balance between antioxidants and oxidants and changes in the concentration of Ca^2+^, as well as the activities of sodium-potassium adenosine triphosphatase (Na^+^/K^+^-ATPase), acetylcholinesterase (AChE), and phospholipase A2 (PLA2) were noted to be involved in the etiopathogenesis of disorders connected with the neurodegeneration [30,31,32]. Moreover, an inhibition of Nrf2 was reported to promote the production of amyloid β, which accumulation in the brain contributes to the development of Alzheimer’s disease [15]. Thus, the impact of Cd via the above pathways in the brain needs to be studied to explain the mechanism of its neurotoxicity and recognize markers of this action under low-to-moderate chronic exposure. This knowledge is necessary for identifying and monitoring the risk of nervous system damage and looking for effective protective and therapeutic strategies.

The studies conducted by our research team in an animal model of current lifetime human exposure to Cd revealed that even a low-level long-term intoxication with this toxic heavy metal creates a risk for health, including damage to the nervous system, whereas the supplementation with an aqueous extract from *Aronia melanocarpa* L. berries ((Michx.) Elliott, Rosaceae; chokeberries) (AE) seems to be an effective protective strategy [20,33,34,35,36,37,38]. Chokeberry extract is a rich source of numerous pro-health compounds, including polyphenols showing strong antioxidative properties, vitamins, and microelements [34,39]. We have already reported that the administration of a 0.1% AE prevented Cd accumulation in the body and protected against this heavy metal-induced damage to the liver, kidneys, and bone, as well as had a favourable influence on the homeostasis of zinc, copper, and manganese in the brain [20,33,34,35,36,37,38]. Moreover, the AE counteracted the Cd-induced oxidative stress, and oxidative damage to lipids, proteins, and deoxyribonucleic acid (DNA) in the brain [20]. Research conducted in a group of healthy people revealed that both short- and long-lasting supplementation with an extract from chokeberries improved the functioning of the nervous system, including the perception and psychomotor functions [40,41]. Moreover, it was reported that the administration of anthocyanins isolated from the berries of *A. melanocarpa* ameliorated learning and memory impairment in rats with amyloid β-induced neurotoxicity [42]. Owing to the available literature data on the outcomes of oxidative stress in the nervous tissue [10,11,12,13,14,15,16,17,18,19,20,21,22,23,24,25], the potential of AE to protect from Cd accumulation [33], and the development of this heavy metal-induced oxidative stress in the brain [20], as well as the neuroprotective activity of *A. melanocarpa* berries products [40,41,42] and natural polyphenols [2], we have hypothesized that this xenobiotic may affect the functioning of the brain even at low-level chronic exposure and that the co-administration of AE may counteract its negative impact. The present study was aimed to verify this hypothesis. To estimate Cd neurotoxicity at low-to-moderate exposure, and whether the supplementation with AE may counteract this action, numerous biomarkers such as chosen proteins, including enzymatic proteins, like AChE, calmodulin (CAM), PLA2, NOS1, Nrf2, KEAP1, Na^+^/K^+^-ATPase, calcium-magnesium adenosine triphosphatase (Ca^2+^/Mg^2+^-ATPase), and chosen MMPs (MMP-2, MMP-3 and MMP-9) and their tissue inhibitors (TIMPs such as TIMP-2, TIMP-3, and TIMP-4), were determined in the aliquots of the brain homogenates. Moreover, the concentrations of total (intracellular and extracellular) calcium (Ca) and magnesium (Mg) in the brain tissue and the activity of AChE in the serum were assayed in the present study. The wide range of measurements allowed the detection of the impact of Cd and AE on the nervous tissue via various pathways. To estimate the involvement of the pro-oxidative action of Cd in its neurotoxicity, as well as the possible role of the antioxidative properties of AE in the mechanism of its protective impact against the neurotoxic action of this toxic heavy metal, mutual dependencies between the measured biomarkers of neurotoxicity and the previously reported [20] main markers of oxidative–reductive status of the brain tissue, such as total oxidative status (TOS), total antioxidative status (TAS), and oxidative stress index (OSI), as well as indices of oxidative damage to proteins (3-nitrotyrosine—3-NT and protein carbonyl groups—PC), lipids (lipid peroxides—LPO and 8-isoprostane—8-iso), and DNA (γ-H2A histone family member X—γ-H2AX), were evaluated. The relationships between biomarkers of neurotoxicity and Cd concentration in the brain, blood, and urine [33] were also estimated. Moreover, mutual dependencies between the measured biomarkers of neurotoxicity were examined. This study is the first to investigate this matter.

## 2. Results

### 2.1. AChE in the Aliquots of the Brain Homogenates and the Serum

**Figure 1:**
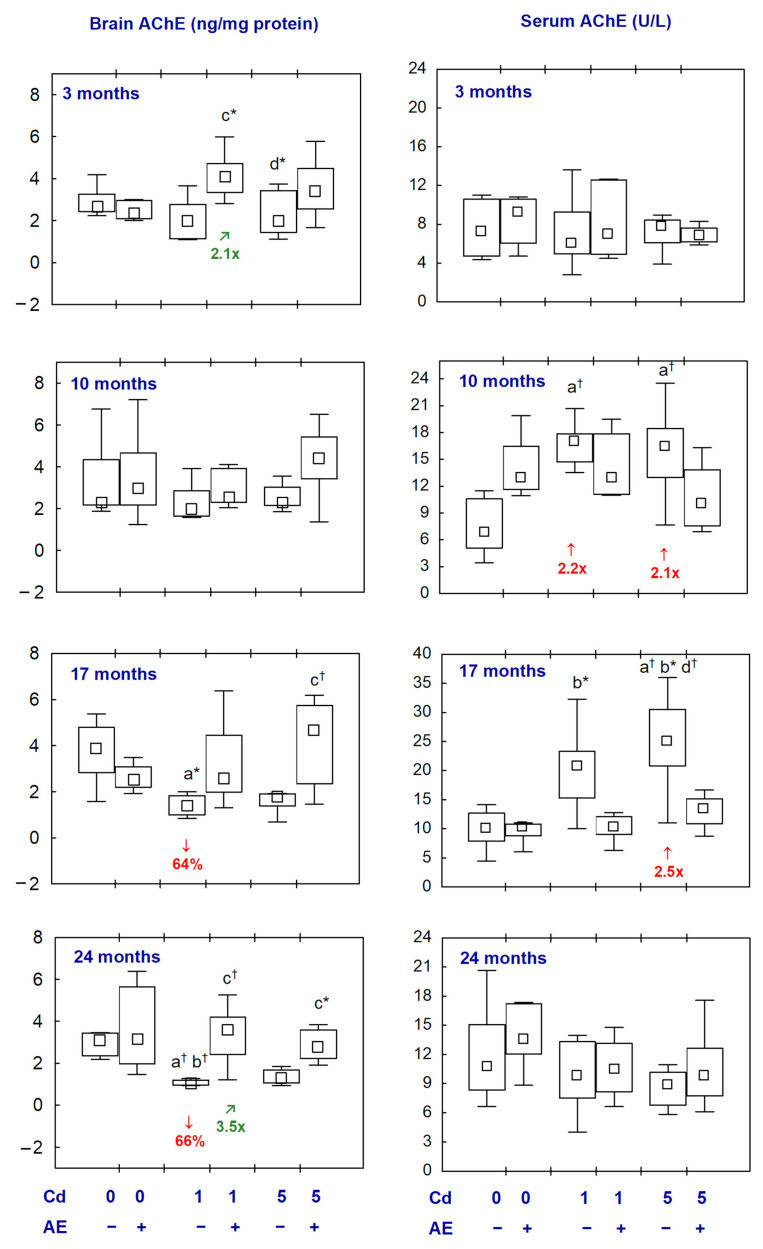
The concentration of acetylcholinesterase (AChE) in the aliquots of the brain homogenates and its activity in the serum of rats administered with cadmium (Cd) at 0, 1, or 5 mg/kg of diet and/or *Aronia melanocarpa* L. berries extract (AE). Different from the: a—Control group, b—AE group, c—Cd_1_ group, and d—Cd_1_ + AE group, where * *p* < 0.05 and ^†^ *p* < 0.01. The values below the bars indicate a percentage difference or a fold of difference in the median values between the respective groups (**↓**, lower than in the control group; **↑**, higher than in the control group; **↗**, higher than in the proper Cd group). The effect size (η^2^) for the differences in the concentration of AChE in the brain and its activity in the serum between the groups was large (0.241–0.555 and 0.349–0.505, respectively). Detailed data on AChE in the brain and serum are available in Table S1.

The administration of AE alone had no impact on the concentration of AChE in the brain and its activity in the serum (Figure 1, Table S1).

The brain concentration of AChE in the Cd_1_ group after 17 and 24 months of the experiment was lower (by 64% and 66%, respectively) than in the control group, whereas, in the Cd_5_ group, it was unchanged at all time points (Figure 1, Table S1). In the Cd_1_ + AE and Cd_5_ + AE groups, the concentration of AChE was within the range of control values throughout the experiment. Moreover, in the Cd_1_ + AE group, the concentration of this enzyme after 3 and 24 months was higher (2.1- and 3.5-fold, respectively) than in the Cd_1_ group (Figure 1, Table S1).

The activity of AChE in the serum in the Cd_1_ group after 10 months and in the Cd_5_ group after 10 and 17 months was higher (from 2.1- to 2.5-fold) compared to the control group (Figure 1, Table S1). In the animals co-administered with Cd and AE, the activity of this enzyme did not differ compared to the control group and the respective groups treated with Cd alone (Figure 1, Table S1).

There were no differences in the values of AChE in the brain and serum between the Cd_1_ and Cd_5_ groups and between the Cd_1_ + AE and Cd_5_ + AE groups at all time points (Figure 1, Table S1).

### 2.2. Na^+^/K^+^-ATPase and Ca^2+^/Mg^2+^-ATPase in the Aliquots of the Brain Homogenates, as Well as Ca and Mg in the Brain

**Figure 2:**
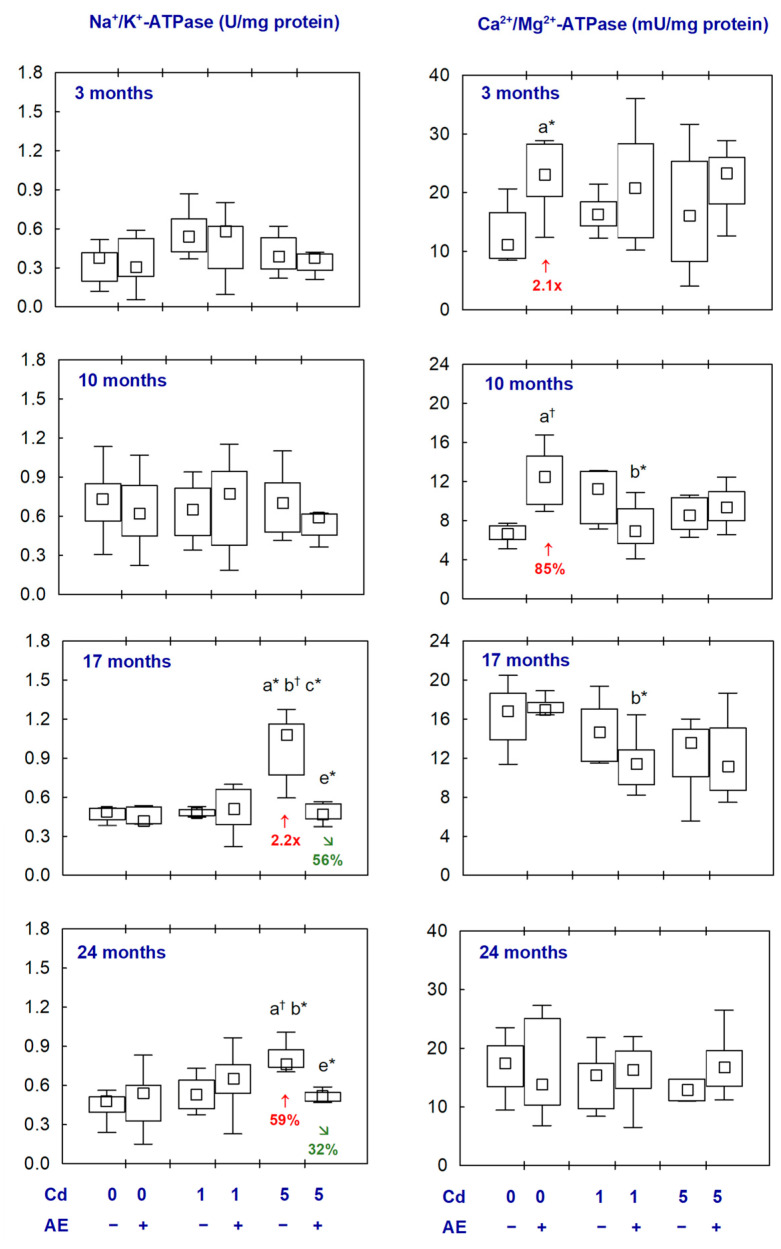
The activities of sodium-potassium adenosine triphosphatase (Na^+^/K^+^-ATPase) and calcium-magnesium adenosine triphosphatase (Ca^2+^/Mg^2+^-ATPase) in the aliquots of the brain homogenates of rats administered with cadmium (Cd) at 0, 1, or 5 mg/kg of diet and/or *Aronia melanocarpa* L. berries extract (AE). Different from the: a—Control group, b—AE group, c—Cd_1_ group, and e—Cd_5_ group, where * *p* < 0.05 and ^†^ *p* < 0.01. The values below the bars indicate a percentage difference or a fold of difference in the median values between the respective groups (****↑****, higher than in the control group; **↘**, lower than in the proper Cd group). The effect size (η^2^) for the differences in the activities of Na^+^/K^+^-ATPase and Ca^2+^/Mg^2+^-ATPase between the groups was large (0.312–0.360 and 0.179–0.334, respectively). Detailed data on the brain activities of Na^+^/K^+^-ATPase and Ca^2+^/Mg2^+^-ATPase are available in Table S2.

**Figure 3:**
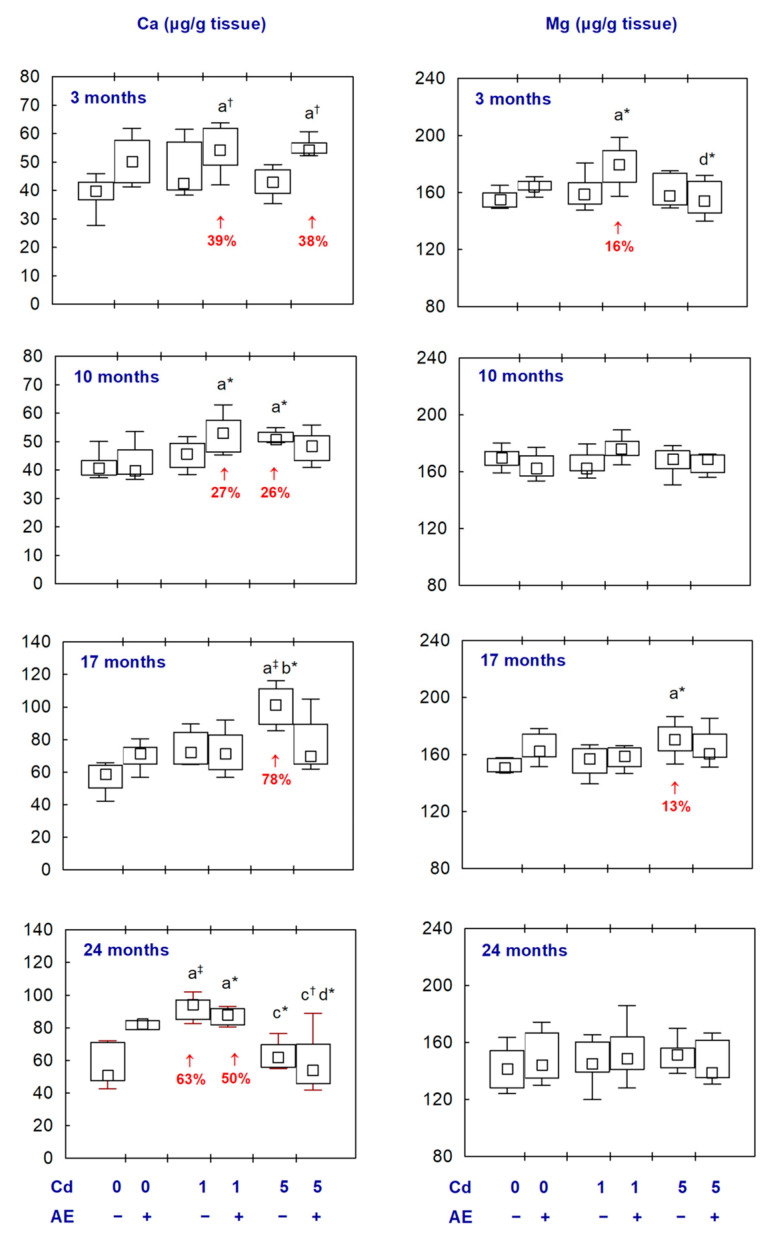
The concentrations of total calcium (Ca) and magnesium (Mg) in the brain of rats administered with cadmium (Cd) at 0, 1, or 5 mg/kg of diet and/or *Aronia melanocarpa* L. berries extract (AE). Different from the: a—Control group, b—AE group, c—Cd_1_ group, and d—Cd_1_ + AE group, where * *p* < 0.05, ^†^ *p* < 0.01, and ^‡^ *p* < 0.001. The values below the bars indicate a percentage difference in the median values between the respective groups (****↑****, higher than in the control group). The effect size (η^2^) for the differences in the concentrations of Ca and Mg between the groups was large or medium (0.322–0.624 and 0.010–0.117, respectively). Detailed data on the concentrations of Ca and Mg are available in Table S3. In Table S3, the total contents of Ca and Mg in the brain are also presented (the directions of changes in these elements content due to the exposure to Cd and co-administration of AE were analogous as in the case of these elements concentrations, except for unchanged Mg content in the Cd_5_ group after 17 months).

The administration of AE alone had no impact on the activities of Na^+^/K^+^-ATPase and Ca^2+^/Mg^2+^-ATPase and the concentrations of Ca and Mg in the brain, except for an increase in the activity of Ca^2+^/Mg^2+^-ATPase after 3 and 10 months (Figure 2 and Figure 3, Tables S2 and S3).

The activity of Na^+^/K^+^-ATPase in the Cd_1_ and Cd_1_ + AE groups did not differ from the control group (Figure 2, Table S2). The exposure to Cd at 5 mg/kg of diet resulted in an increase in this enzyme activity after 17 and 24 months (2.2-fold and by 59%, respectively); however, in the case of co-administration of AE, the activity did not differ from the control group (Figure 2, Table S2). The exposure to Cd at both concentrations in the diet with or without AE supplementation did not influence the activity of Ca^2+^/Mg^2+^-ATPase in the brain (Figure 2, Table S2).

The brain concentration of Ca in the Cd_1_ group after 3–17 months did not differ compared to the control group; however, in the case of the co-administration of AE (Cd_1_ + AE group), it was enhanced after 3 and 10 months (by 39% and 27%, respectively). After 24 months, the concentration of this element in the Cd_1_ group and Cd_1_ + AE group was higher (by 63% and 50%, respectively) than in the control animals (Figure 3, Table S3). In the Cd_5_ group, the concentration of Ca after 10 and 17 months was increased (by 26% and 78%, respectively) but, in the animals co-administered with AE, it did not differ compared to the control group. Moreover, the 3-month administration of AE to the animals maintained on a diet containing Cd at the concentration of 1 and 5 mg/kg enhanced (by 39% and 38%, respectively) the Cd alone-unaffected concentration of this bioelement (Figure 3, Table S3).

The treatment with Cd alone had no impact on the concentration of Mg in the brain except for its increase (by 13%) at the moderate exposure after 17 months. In the animals co-administered with Cd and AE, the concentration of this element did not differ compared to the respective group treated with Cd alone and the control group, except for its increased (by 16%) value in the Cd_1_ + AE group after 3 months (Figure 3, Table S3).

There were no differences in the above parameters between the Cd_1_ and Cd_5_ groups and between the Cd_1_ + AE and Cd_5_ + AE groups, except for a higher activity of Na^+^/K^+^-ATPase in the Cd_5_ group after 17 months, as well as a lower concentration of Ca after 24 months in the Cd_5_ group than in the Cd_1_ group and in the Cd_5_ + AE group than in the Cd_1_ + AE group and lower concentration of Mg after 3 months in the Cd_5_ + AE group as compared to the Cd_1_ + AE group (Figure 2 and Figure 3, Tables S2 and S3).

### 2.3. CAM, PLA2, and NOS1 in the Aliquots of the Brain Homogenates

**Figure 4:**
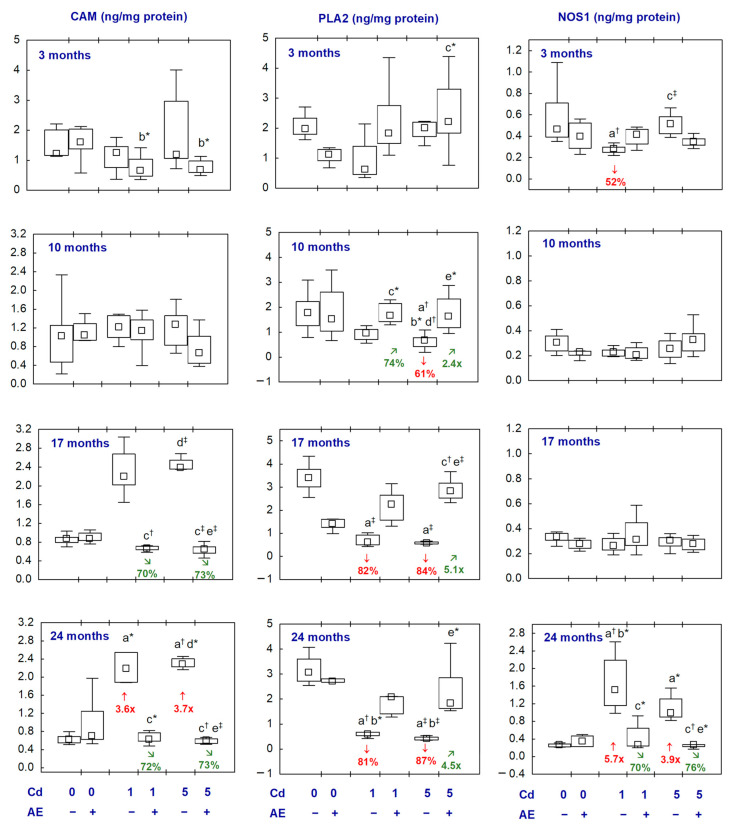
The concentrations of calmodulin (CAM), phospholipase A2 (PLA2), and nitric oxide synthase 1 (NOS1) in the aliquots of the brain homogenates of rats administered with cadmium (Cd) at 0, 1, or 5 mg/kg of diet and/or *Aronia melanocarpa* L. berries extract (AE). Different from the: a—Control group, b—AE group, c—Cd_1_ group, d—Cd_1_ + AE group, and e—Cd_5_ group, where * *p* < 0.05, ^†^ *p* < 0.01, and ^‡^ *p* < 0.001. The values below the bars indicate a percentage difference or a fold of difference in the median values between the respective groups (****↑****, higher than in the control group; ****↓****, lower than in the control group; ****↗****, higher than in the proper Cd group; **↘**, lower than in the proper Cd group). The effect size (η^2^) for the differences in CAM, PLA2, and NOS1 concentrations between the groups was large (0.327–0.818, 0.276–0.838, and 0.403–0.575, respectively). Detailed data on CAM, PLA2, and NOS1 concentrations are available in Table S4.

The administration of AE alone did not influence the concentrations of CAM, PLA2, and NOS1 in the brain (Figure 4, Table S4).

The concentration of CAM in the Cd_1_ and Cd_5_ groups was increased (3.6- and 3.7-fold, respectively) after 24 months (Figure 4, Table S4). In the Cd_1_ + AE and Cd_5_ + AE groups, the concentration of this parameter did not differ compared to the control group throughout this study and, after 17 and 24 months, it was lower (by 70–73%) than in the appropriate Cd groups (Figure 4, Table S4).

The concentration of PLA2 in the brain in the Cd_1_ group after 17 and 24 months and in the Cd_5_ group after 10, 17, and 24 months was lower (by 61–87%) compared to the control group. In the animals supplemented with AE during the treatment with Cd, the concentration of PLA2 throughout this study was within the values detected in the control group. Moreover, at some time points, it was higher than in the appropriate group treated with Cd alone (Figure 4, Table S4).

The concentration of NOS1 was lower (by 52%) after the exposure to Cd at 1 mg/kg of diet for 3 months, whereas after 24 months at both levels of the treatment, it was higher (5.7- and 3.9-fold, respectively) (Figure 4, Table S4). In the Cd_1_ + AE and Cd_5_ + AE groups, the concentration was within the range of values noted in the control animals, and after 24 months it was lower (by 70% and 76%, respectively) than in the appropriate Cd groups (Figure 4, Table S4).

There were no differences between the Cd_1_ and Cd_5_ groups and between the Cd_1_ + AE and Cd_5_ + AE groups in the concentrations of CAM, NOS1, and PLA2 (Figure 4, Table S4).

### 2.4. Nrf2 and KEAP1 in the Aliquots of the Brain Homogenates

**Figure 5:**
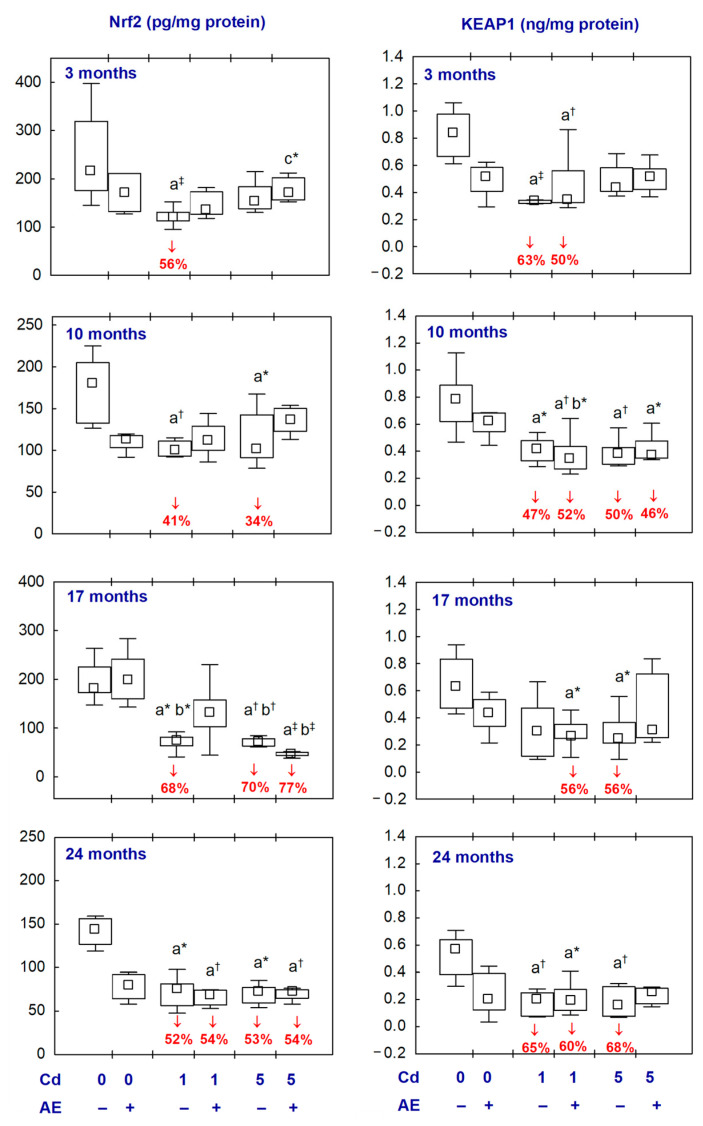
The concentrations of nuclear factor erythroid 2-related factor 2 (Nrf2) and Kelch-like ECH-associated protein 1 (KEAP1) in the aliquots of the brain homogenates of rats administered with cadmium (Cd) at 0, 1, or 5 mg/kg of diet and/or *Aronia melanocarpa* L. berries extract (AE). Different from the: a—Control group, b—AE group, and c—Cd_1_ group, where * *p* < 0.05, ^†^ *p* < 0.01, and ^‡^ *p* < 0.001. The values below the bars indicate a percentage difference in the median values between the respective groups (****↓****, lower than in the control group). The effect size (η^2^) for the differences in the concentrations of Nrf2 and KEAP1 between the groups was large (0.383–0.723, and 0.248–0.495, respectively). Detailed data on Nrf2 and KEAP1 concentrations are available in Table S5.

There were no differences in the brain concentrations of Nrf2 and KEAP1 between the AE and control groups (Figure 5, Table S5).

The concentration of Nrf2 in the Cd_1_ and Cd_5_ groups at all time points (except for its unaffected concentration in the Cd_5_ group after 3 months), Cd_1_ + AE group after 24 months, and the Cd_5_ + AE group after 17 and 24 months was lower (from 34% to 77%) than in the control group (Figure 5, Table S5).

The treatment with Cd at 1 and 5 mg/kg of diet resulted in a decrease (from 47% to 68%) in the concentration of KEAP1, except for the Cd_1_ group after 17 months and the Cd_5_ group after 3 months in which this parameter was unaffected (Figure 5, Table S5). In the Cd_1_ + AE group at all time points and in the Cd_5_ + AE group after 10 months, the concentration of KEAP1 was also lower (from 46% to 60%) compared to the control group and it did not differ compared to the respective groups receiving Cd alone (Figure 5, Table S5). In the animals co-administered with Cd in the diet at the concentration of 5 mg/kg and AE for 3, 17, and 24 months, the concentration of KEAP1 did not differ from the control group (Figure 5, Table S5).

The values of Nrf2 and KEAP1 did not differ between the Cd_1_ and Cd_5_ groups and between the Cd_1_ + AE and Cd_5_ + AE groups at all time points (Figure 5, Table S5).

### 2.5. MMPs and TIMPs in the Aliquots of the Brain Homogenates

**Figure 6:**
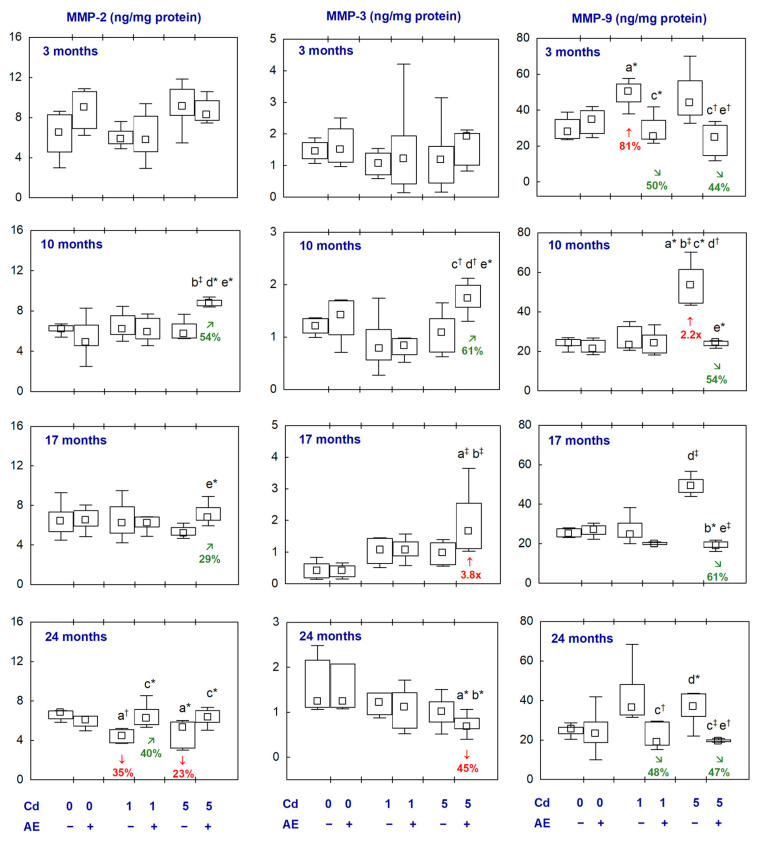
The concentrations of metalloproteinases (MMPs) such as MMP-2, MMP-3, and MMP-9 in the aliquots of the brain homogenates of rats administered with cadmium (Cd) at 0, 1, or 5 mg/kg of diet and/or *Aronia melanocarpa* L. berries extract (AE). Different from the: a—Control group, b—AE group, c—Cd_1_ group, d—Cd_1_ + AE group, and e—Cd_5_ group, where * *p* < 0.05, ^†^ *p* < 0.01, and ^‡^ *p* < 0.001. The values below the bars indicate a percentage difference or a fold of difference in the median values between the respective groups (****↑****, higher than in the control group; ****↓****, lower than in the control group; ****↗****, higher than in the proper Cd group; **↘**, lower than in the proper Cd group). The effect size (η^2^) for the differences in MMP-2, MMP-3, and MMP-9 concentrations between the groups was medium-to-large (0.128–0.451, 0.289–0.546, and 0.388–0.683, respectively). Detailed data on the concentrations of determined MMPs are available in Table S6.

**Figure 7:**
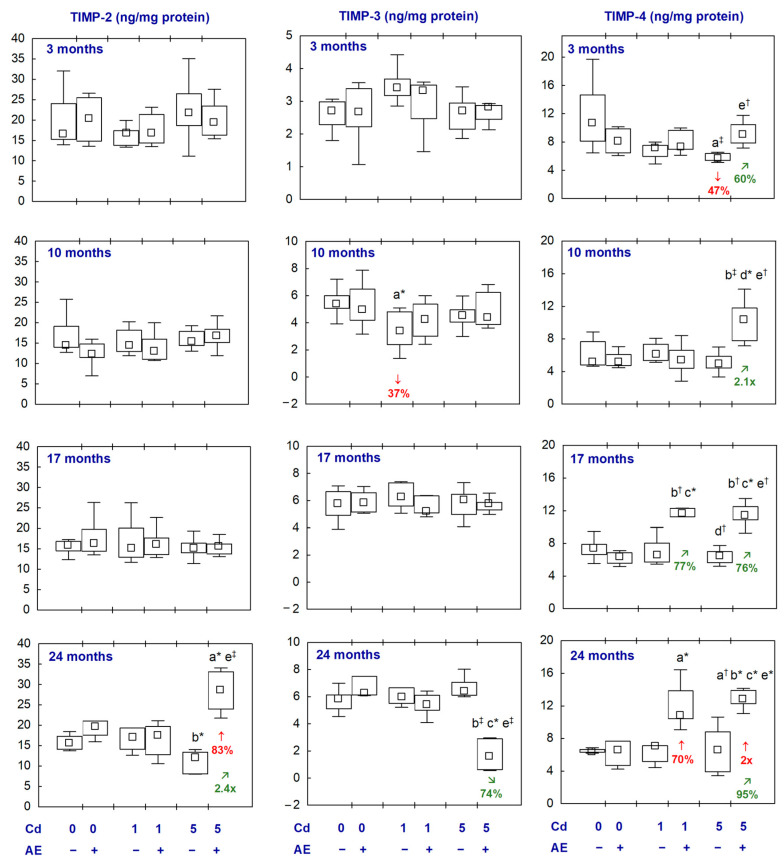
The concentrations of tissue inhibitors of metalloproteinases (TIMPs) such as TIMP-2, TIMP-3, and TIMP-4 in the aliquots of the brain homogenates of rats administered with cadmium (Cd) at 0, 1, or 5 mg/kg of diet and/or *Aronia melanocarpa* L. berries extract (AE). Different from the: a—Control group, b—AE group, c—Cd_1_ group, d—Cd_1_ + AE group, and e—Cd_5_ group, where * *p* < 0.05, ^†^ *p* < 0.01, and ^‡^ *p* < 0.001. The values below the bars indicate a percentage difference or a fold of difference in the median values between the respective groups (****↑****, higher than in the control group; ****↓****, lower than in the control group; ****↗****, higher than in the proper Cd group; **↘**, lower than in the proper Cd group). The effect size (η^2^) for the differences in TIMP-2, TIMP-3, and TIMP-4 concentrations between the groups was medium-to-large (0.566, 0.131–0.531, and 0.354–0.611, respectively). Detailed data on the concentrations of determined TIMPs are available in Table S7.

The administration of AE alone did not influence the concentrations of the examined MMPs and TIMPs in the brain (Figure 6 and Figure 7, Tables S6 and S7).

In the animals exposed to Cd at 1 and 5 mg/kg of diet, the concentration of MMP-3 was unaffected throughout the whole experiment and MMP-2 concentration was decreased (by 35% and 23%, respectively) after 24 months. The concentration of MMP-9 was increased in the Cd_1_ group after 3 months (by 81%) and, after 10 months in the Cd_5_ group, reached a median value higher (2.2-fold) compared to the control group (Figure 6, Table S6). In the groups supplemented with AE under the treatment with Cd, the concentrations of the determined MMPs did not differ from the control group, except for the concentration of MMP-3 in the Cd_5_ + AE group which was higher (3.8-fold) after 17 months and lower (by 45%) after 24 months (Figure 6, Table S6). Moreover, at some time points, there were differences in the concentration of particular MMPs between the group co-administered with Cd and AE and the proper group treated with Cd alone; however, these concentrations in the case of co-administration of Cd and AE did not differ compared to the control group (Figure 6, Table S6).

The treatment with Cd had no impact on the concentrations of the examined TIMPs in the brain, except for a temporary decrease (by 37%) in the concentration of TIMP-3 in the Cd_1_ group (after 10 months) and in the concentration of TIMP-4 in the Cd_5_ group (after 3 months) (Figure 7, Table S7). In the animals supplemented with AE under the exposure to Cd, the concentrations of TIMPs did not differ compared to the control group, except for increased concentrations of TIMP-2 in the Cd_5_ + AE group (by 83%) and TIMP-4 in the Cd_1_ + AE and Cd_5_ + AE groups (70% and 2-fold, respectively) after 24 months (Figure 7, Table S7).

There were no differences between the values of particular MMPs and TIMPs between the Cd_1_ and Cd_5_ groups, as well as between the Cd_1_ + AE and Cd_5_ + AE groups, except for a higher concentration of MMP-9 in the Cd_5_ group than in the Cd_1_ group and the concentrations of MMP-2, MMP-3, and TIMP-4 in Cd_5_ + AE group than in the Cd_1_ + AE group after 10 months (Figure 6 and Figure 7, Tables S6 and S7).

### 2.6. Relationships between the Determined Markers of Neurotoxicity and Cd Concentration in the Brain, Blood, and Urine

In the animals that were not supplemented with AE (the control group, Cd_1_ group, and Cd_5_ group) there were negative correlations between the concentrations of AChE, Nrf2, KEAP1, and TIMP-4 in the brain and the concentration of Cd in the brain, blood, and urine (Table 1). The concentrations of CAM and MMP-9 positively correlated with these concentrations of Cd (Table 1). Moreover, positive correlations were noted between Cd concentration in the brain and the activity of Na^+^/K^+^-ATPase and the concentration of Mg, as well as between the brain Ca concentration and Cd concentration in the blood and urine (Table 1).

In the female rats administered with AE (the AE group, Cd_1_ + AE group, and Cd_5_ + AE group), the concentration of CAM and MMP-9 correlated negatively, and the concentration of TIMP-4 correlated positively with the concentration of Cd in the brain, blood, and urine (Table 1). Moreover, positive relationships occurred between the concentration of MMP-2 and Cd concentration in the blood and urine, as well as between the concentrations of AChE and NOS1 and Cd concentration in the brain, whereas between the concentrations of TIMP-3 and Cd in the brain and urine negative dependencies were noted (Table 1).

All the above-described dependencies were weak to moderate (|r| = 0.217–0.572) (Table 1).

### 2.7. Relationships between the Determined Markers of Neurotoxicity and TOS, TAS, and OSI in the Aliquots of the Brain Homogenates

In the rats not supplemented with AE, negative relationships were noted between the concentrations of AChE, Nrf2, KEAP1, and TIMP4 and the values of TOS and OSI in the brain. Positive dependencies occurred between the brain tissue TOS and OSI and the concentrations of CAM and MMP-9 (Table 2). The brain OSI positively correlated with the activity of Na^+^/K^+^-ATPase and the concentrations of TIMP-3 and Ca, and negatively with the concentrations of PLA2 and MMP-2 (Table 2). Moreover, the brain TAS positively correlated with the concentrations of AChE, Mg, PLA2, Nrf2, KEAP1, MMP-2, and TIMP-2, and negatively with the activity of Na^+^/K^+^-ATPase and the concentrations of TIMP-3 and Ca (Table 2).

In the animals treated with AE, there were correlations between TOS and TAS and the concentrations of TIMP-3 and Ca (negative dependencies), and the concentrations of Mg, Nrf2, and MMP-3 (positive dependencies) in the brain (Table 2). Positive relationships were noted between the concentration of MMP-2 and TOS, the value of TAS and the activity of Ca^2+^/Mg^2+^-ATPase, and the concentrations of NOS1, KEAP1, and MMP-9, as well as between the concentration of TIMP-4 and OSI in the brain (Table 2). Moreover, negative relationships occurred between TAS and the concentrations of PLA2 and TIMP-4, and between OSI and the activity of Ca^2+^/Mg^2+^-ATPase in the brain (Table 2).

### 2.8. Relationships between the Determined Markers of Neurotoxicity and Indices of Oxidative Modifications of Proteins, Lipids, and DNA in the Aliquots of the Brain Homogenates

In the female rats not administered with AE, some dependencies were noted between the determined markers of neurotoxicity and indices of oxidative damage to proteins (3-NT and PC), lipids (LPO and 8-iso), and DNA (γ-H2AX) (Table 3). The most important among these relationships (weak to moderate) are negative correlations between KEAP1 and Nrf2 and markers of oxidative damage of cellular macromolecules, such as PC, LPO, 8-iso, and γ-H2AX. An inverse association was also noted between PLA2 and 8-iso. The brain concentration of AChE also negatively correlated with the concentrations of 3-NT, PC, LPO, 8-iso, and γ-H2AX. Moreover, a positive dependence occurred between the concentration of NOS1 and the concentrations of 3-NT, PC, LPO, 8-iso, and γ-H2AX in the nervous tissue (Table 3).

In the animals supplemented with AE (the AE, Cd_1_ + AE, and Cd_5_ + AE groups), positive dependencies were noted between Nrf2 and KEAP1 concentrations and the concentrations of 3-NT, LPO, and γ-H2AX (Table 3).

### 2.9. Mutual Relationships between the Determined Markers of Neurotoxicity

There were positive and negative dependencies between some of the examined parameters in the brains of rats administered (the AE, Cd_1_ + AE, and Cd_5_ + AE groups) or not administered with AE (the control, Cd_1_, and Cd_5_ groups) and they are presented in Table 4. Among others, a positive dependence occurred between Nrf2 and KEAP1 in the animals not supplemented and supplemented with AE. Moreover, positive dependencies between chosen MMPs and TIMPs were noted in the rats administered with AE (Table 4).

In both the animals not administered with AE and those supplemented with the extract, there was no dependency between the brain concentration of AChE and the serum activity of this enzyme (r = −0.148, *p* = 0.17 and r = 0.003, *p* = 0.98, respectively).

## 3. Discussion

The present study is the first report that pointed out that long-lasting low-to-moderate exposure to Cd had a damaging impact on the nervous system reflected in changed values of important biomarkers of neurotoxicity, including enzymes and non-enzymatic proteins crucial for the functioning of the nervous system, as well as that the concomitant supplementation with AE had a protective impact against these effects. Because this study was performed in the experimental rat’s model of current environmental human exposure to Cd [33], the findings may be extrapolated into humans; it seems possible that the present exposure to this xenobiotic in developed countries may contribute to the malfunction of the brain. This study confirmed our previous assumption that the no observed adverse effect level (NOAEL) of exposure to Cd for damage to the nervous system is lower than 39.2–83.8 μg/kg body weight (b.w.) (daily doses of Cd during the 24-month feeding with the diets containing this element at the concentration of 1 mg/kg) [20], and provided important data to explain the potential mechanisms of the neurotoxic action of this heavy metal at low-to-moderate exposure. Moreover, this study indicated that chokeberry products may be a preventive strategy against the Cd-induced damage to the nervous tissue, and it allowed the proposal of the possible mechanism of the beneficial action.

The brain is partially protected from the access of harmful substances, including toxic metals, via the blood–brain barrier (BBB) [9,43]; however, it is known that the barrier can be damaged by different factors (inclusive of Cd itself) [9,44]. These let this xenobiotic to accumulate and exert toxic effects on this organ [14,20]. It is has been shown that the mechanism of the neurotoxic action of Cd is connected with the indirect induction of oxidative stress in the nervous tissue [10,11,12,13,14,15,16,17,18,19,20,21,22,23,24,25]. Our recent measurements [20] performed in the aliquots of homogenates of the brain used in the current study revealed that this xenobiotic may exert pro-oxidative action in the nervous tissue even at low-level exposure and low accumulation in this organ. Cd inhibited the brain activities of antioxidative enzymes (superoxide dismutase—SOD, catalase—CAT, glutathione reductase—GR, and glutathione peroxidase—GPx) and declined the concentration of non-enzymatic antioxidants leading to a decrease in TAS of the nervous tissue, which is the main marker of the ability to antioxidative response [20]. Moreover, the exposure increased TOS, due to enhanced concentrations of pro-oxidants (including hydrogen peroxide, myeloperoxidase, and xanthine oxidase), and resulted in a disturbed balance between the processes of oxidation and reduction in the brain with the predominance of the former leading to the development of oxidative stress (reflected in increased values of OSI; noted throughout this study at both levels of exposure except for the Cd_1_ group after 3 months) with enhanced lipid peroxidation and oxidative damage to proteins and DNA in the nervous tissue [20]. As has been noted in the present study, the changed concentrations or activities of various biomarkers of neurotoxicity, including enzymes such as AChE, Na^+^/K^+^-ATPase, PLA2, and NOS1, non-enzymatic proteins such as CAM, Nrf2, and KEAP1, as well as Ca, and some MMPs (MMP-2 and MMP-9) and TIMPs (TIMP-3 and TIMP-4), show that Cd can damage the nervous system also via other mechanisms. It is important to note that the changes of some of the parameters connected with oxidative stress (Nrf2, KEAP1, as well as MMP-9 and TIMP-4) occurred as early as after 3 months of exposure to this xenobiotic at the first dysregulation of the redox balance (decrease in TAS at low exposure and increase in OSI due to the moderate treatment) [20]. However, it is worth paying attention to the fact that the pro-oxidative effect of Cd could underlie these mechanisms and favour its action by these pathways.

One of the pathomechanisms of Cd neurotoxicity at low-to-moderate exposure can involve a decrease in the concentrations of Nrf2 and KEAP1. Normally KEAP1 inactivates Nrf2 [15,22,23,26]; so, the simultaneous decline in the concentrations of both parameters in the brain under the exposure to Cd at 1 and 5 mg/kg of diet and the positive correlation between these variables seem to indicate an uncoupling of these parameters. Moreover, the negative relationships between Cd concentration in the brain, blood, and urine and the brain concentrations of KEAP1 and Nrf2 indicate that the uncoupling will intensify with growing exposure and enhanced accumulation of this xenobiotic in the brain. The negative dependencies between these two parameters (KEAP1 and Nrf2) and TOS, OSI, and markers of oxidative damage to cellular macromolecules in the brain, such as PC, LPO, 8-iso, and γ-H2AX, show that this uncoupling will also intensify with the increasing severity of oxidative damage to the nervous tissue. A decrease in the concentration of Nrf2 in the brain can cause the impairment of the “Nrf2-antioxidant response elements signalling” (Nrf2-ARE) and lead to the development of oxidative stress via disturbations in the regulation of the expression of phase II detoxification genes and antioxidants [22,23,26,45]. The available literature data concerning the direction of changes in the values of Nrf2 and KEAP1 due to exposure to Cd are inconsistent; however, both an elevation and reduction in these parameters are connected with damage to the nervous system [15,22,23,26].

The noted increase in the concentration of NOS1 after the 24-month exposure to both Cd levels confirmed oxidative damage caused by this xenobiotic in the nervous tissue of these animals described in the recent report by us [20]. The physiological role of NOS1 is to synthesize nitric oxide (NO), which works as a second neurotransmitter in synapses [46,47]. The primary role of NO in the brain is to increase the level of cyclic guanosine 3′,5′-monophosphate (c-GMP) in the nervous cells and, in this way, influence the cGMP-dependent signalling pathway, involved in the regulation of learning and other complex behaviours [47]. An elevated concentration of NO leads to the induction of nitrosative stress resulting in the S-nitrosylation of peroxiredoxin 2 that regulates the function, structure, and survival of the nervous cells. Moreover, nitrosative stress is engaged in the development of diseases connected with neuroinflammation and neurodegeneration, such as Alzheimer’s disease or Parkinson’s disease [46]. Elevated NO levels are noted in various states associated with oxidative imbalance such as the processes of neuroinflammation or neurodegeneration [46]. The increase in the concentration of NOS1 in the brain, noted in the present study, at both levels of exposure to Cd and the positive relationship between the concentrations of NOS1 and 3-NT (a specific marker of oxidative damage to the protein-bound and free tyrosine induced by reactive nitrogen species) suggest that the concentration of NO in the brain tissue might also be elevated. Thus, it seems possible that the mechanism of Cd neurotoxicity may also involve the pathways connected with excessive production and action of NO. The activation of NOS1, with a subsequent increase in NO synthesis, might be stimulated by the signalling dependent on Ca^2+^ and CAM [47]. Under physiological conditions there is a positive dependence between NOS1 and Nrf2 [48], the decrease in the concentration of NOS1 noted after 3 months of low-level exposure, when oxidative stress has not yet occurred, might be connected with the simultaneously decreased concentration of Nrf2. Considering our previous findings on the oxidative–antioxidative status of the nervous tissue [20], an increase in the concentration of NOS1 might be expected; however, the decrease in the concentration of this enzyme in the Cd_1_ group after 3 months is an unexpected result.

The modifying impact of the exposure to Cd on the concentrations of AChE and CAM in the brain, engaged in the regulation of the release of neurotransmitters, shows that chronic low-to-moderate exposure to this heavy metal caused disturbances in neurotransmission. The primary role of AChE is the termination of impulse transmission at cholinergic synapses by the rapid breaking down (hydrolysis) of acetylcholine and certain other choline esters that act as neurotransmitters [49]. Thus, the reduction in this enzyme concentration in the brain caused by Cd might result in the persistence of an elevated level of acetylcholine within synapses and lead to increased cholinergic signalling within the central nervous system [49]. The finding that the concentration of AChE in the brain, after the 17- and 24-month treatment with Cd at 1 mg/kg of diet alone, was decreased indicates that long-term low-level exposure to this xenobiotic may lead to cholinergic hyperactivity. It is worth noting that, although in the Cd_5_ group, the activity of AChE in the brain did not differ compared to the control group, a tendency to decline after 17 and 24 months was noted (*p* = 0.06 and *p* = 0.07, respectively). The negative dependencies between the concentration of AChE and markers of oxidative status of the brain (TOS, OSI, 3-NT, PC, LPO, 8-iso, and γ-H2AX suggest that the Cd-induced cholinergic hyperactivity may intensify with the growing extent of oxidative stress in the nervous tissue. The results of other authors concerning the impact of Cd on the activity of AChE in the brain are inconsistent as both an increased and decreased activity of this enzyme was reported [11,12,13,14,18,19,21]. Moreover, data from other authors indicated a Cd-induced enhancement in the activity of AChE in the whole blood and leukocytes [50] and a decrease in its serum activity [21] but, in our study, the serum activity of this enzyme was increased. The interesting finding of the present research is a lack of dependence between AChE concentration in the brain and its activity in the serum. It is important to emphasize that there was no simultaneous change in AChE in the brain and serum. In the case when the brain concentration of AChE was decreased the activity of this enzyme in the serum was unaffected. If the activity of AChE in the serum was increased its brain concentration remained unchanged. Our results indicate that the assay of AChE in the serum does not reflect the brain status of this enzyme during low-to-moderate exposure and may stay proper despite its decreased level in the brain (by even 64–66%).

The Cd-induced enhancement in the concentration of Ca in the brain may indicate that this toxic heavy metal might impact the homeostasis of Ca in the nervous tissue and thus influence the concentration of CAM, the protein regulating the release of signalling molecules, including second messengers such as Ca^2+^. This protein decreases Ca^2+^ concentration inside the nervous cells via activating calcium adenosine triphosphatase (Ca^2+^-ATPase) [17,25]. Therefore, it seems possible that the enhanced concentration of CAM in the brain after a long-term treatment with Cd might be an adaptative response of the nervous tissue to this heavy metal-induced elevation of Ca concentration. The improper concentration of Ca in the brain can result in the dysfunction of this organ, including the dysregulation of the differentiation of cells and their metabolism [17,25]. The results of our study regarding CAM concentration in the brain are consistent with data by other authors [17]. Ca^2+^/Mg^2+^-ATPase is also involved in maintaining an appropriate concentration of Ca^2+^ in the cell [17]; however, its activity in the present study was not upregulated under exposure to Cd. These might be related, at least to some extent, to a proper concentration of Mg in the brain throughout the whole study at low-level exposure and with only the transient (after 17 months) increase at the moderate treatment. Unlike in our research, a decrease in the concentration of Mg [51] and the activity of Ca^2+^/Mg^2+^-ATPase [25] were noted by other researchers; however, it was due to subacute exposure to Cd (18 mg Cd/kg b.w. for 14 days and 10 mg Cd/kg b.w. for 21 days), which can indicate that Cd acts contrarily during different intensity of exposure and its duration.

The Cd-induced activation of Na^+^/K^+^-ATPase shows that the homeostasis of ions such as sodium (Na^+^) and potassium (K^+^) was disturbed in the nervous cells. Na^+^/K^+^-ATPase regulates the flow of these ions to the inside and outside of the cell [52,53]. Physiologically, this enzyme leads to the import of two K^+^ to the cell and the export of three Na^+^, so its elevated activity can lead to an excess of K^+^ inside the cells and a deficiency of Na^+^. The results of other authors concerning the brain activity of Na^+^/K^+^-ATPase under exposure to Cd are inconsistent as their increase and decrease were noted [11,12,18,19,50].

An important finding of our study is also revealing that the damaging impact of Cd on the nervous system, even at low-level exposure, may be associated with a decrease in the concentration of PLA2 in the brain. This enzyme is responsible for lipid metabolism. It hydrolyses glycerophospholipids in the cellular membranes to release free fatty acids, including arachidonic acid and lysophospholipids, which can act as second messengers in the central nervous system [54]. Moreover, PLA2 is engaged in membrane remodelling and generating proinflammatory mediators, including leukotrienes and prostaglandins [54]. Thus, the decrease in the concentration of PLA2 noted in the current study might result in an improper inflammatory response and lipid homeostasis in the brain. It may also be supported by the fact that a negative correlation between the concentrations of PLA2 and 8-iso in the brain occurred. Sivaprakasam et al. [54] reported an elevation of PLA2 level and a decrease in the concentration of phospholipids in the brain of rats due to acute exposure to Cd; however, in the available literature, there is no data concerning long-lasting exposure.

The results of the present study show that another possible mechanism of the neurotoxic action of Cd under low-to-moderate exposure is its influence on the structure and function of matrix macromolecules in the brain [55] via modification of the concentrations of MMPs and TIMPs. Physiologically, MMPs are engaged in processes such as remodelling neural networks, communication between the neurons and glial cells, and regulating the functioning of the BBB [56]. As the oxidative–antioxidative imbalance activates MMPs [28], and exposure to Cd resulted in the development of oxidative stress in the brain, this pro-oxidative metal might impact their levels, at least partially, in this way. Enhanced concentrations of chosen MMPs (including MMP-9) in the cerebrospinal fluid were reported in numerous states, such as Alzheimer’s disease, dementia, and multiple sclerosis [57]. Moreover, taking into consideration the fact that the increased activity of MMP-9 can contribute to the damage to the BBB [55], the finding that even a short-term exposure to Cd increased the concentration of MMP-9 seems to have particular importance in understanding the mechanism by which this xenobiotic can cross the barrier and reach the brain. Our results agree with the data of other researchers who reported that Cd increased the activity/expression of MMP-9 and decreased the activity of MMP-2 in the brain; however, their findings referred to higher levels or/and shorter duration of exposure [24,58].

Although numerous experimental studies carried out in different animal models provided pieces of evidence that exposure to Cd can influence various biomarkers of neurotoxicity, they have limited value in the context of extrapolating their results on the potential effects that can be noted in humans under long-lasting environmental exposure, as high doses of Cd and/or short-term intoxication were investigated [11,12,13,14,16,17,18,19,21,22,23,24,25,26,50,51,58]. The lowest dose of Cd given to rats in these studies was 1 μg/kg b.w. [58]. This dose is markedly lower than in our study; however, the exposure lasted only 37 days, so it was impossible to investigate the long-term effect of the action of this xenobiotic. The measurements of numerous markers of neurotoxicity performed in the present study revealed that moderate and even low-level exposure to Cd can influence the proper functioning of the brain. The Cd-induced changes in the measured parameters in the brain proved that this xenobiotic might impair the functioning of the nervous system, as it influenced proteins engaged in the regulation of redox homeostasis, release of neurotransmitters, homeostasis of ions, differentiation of cells, or functioning of the BBB [16,17,22,26,45,56].

The noting in our experimental model that the most numerous changes in the values of the measured markers of neurotoxicity after 17 and 24 months (the 24-month experiment duration reflects about 60–70 years of human lifespan) of the treatment with Cd shows that the risk of nervous system damage due to low-to-moderate exposure to this xenobiotic increases with exposure duration. However, some of these variables were affected already after 3 months, indicating that even exposure in youth may have a negative, although very subtle, impact on the nervous system. Although an occurrence of the effect of Cd on the values of some parameters depended on the level of exposure and its duration, the lack of differences in the values of most of the parameters examined in the present study between low-level and moderate exposure to Cd at specific time points might be caused by no difference in the accumulation of this metal in the brain between these groups [33]. It may suggest that the risk of damage to the nervous system at low-level exposure to Cd may be similar to the moderate treatment. Although the measurements of biomarkers of neurotoxicity performed in the present study provided unquestionable evidence for the harmful action of this xenobiotic even at low exposure, the changes in the examined parameters have not yet caused any disturbances in the functioning of the nervous system that could be detected in everyday clinical observations of the animals during the experiment [20]. This should not be a surprise, because the level of exposure to Cd was low or moderate. Moreover, in our study, this xenobiotic was the only neurotoxic factor. The situation in human life is completely different. Humans are usually simultaneously exposed to more factors that can adversely affect the nervous system [3,4,8] and the effects of their actions can be intensified by synergistic interactions, or at least summing up. Therefore, the demonstration that chronic low exposure to Cd during a lifespan adversely affects the processes occurring in the nervous tissue indicates the need to pay more attention to environmental exposure to this toxic heavy metal as one of the risk factors for the increasing incidence of diseases of the nervous system, including neurodegenerative diseases in highly developed countries [3,4].

The finding that even low-level exposure to Cd may create a risk of nervous system damage and the recognition of some mechanisms of this action are important outcomes of the present study facing unavoidable general population lifetime exposure to this xenobiotic [5,6,7] and the growing incidence of nervous system diseases [3,4]. The next significant and, in our opinion, the most important achievement of the study was the finding that the administration of AE had a protective impact against the neurotoxic action induced by chronic low-to-moderate exposure to Cd. The finding that in the animals supplemented with AE under exposure to this toxic heavy metal, the values of numerous parameters variously modified (decreased or increased compared to the proper values) by Cd alone at particular time points (such as the activity of Na^+^/K^+^-ATPase and the concentrations of AChE, CAM, NOS1, PLA2, MMP-2, MMP-9 and TIMP-3 in the brain) throughout the whole study were within the range of values noted in the control group shows that administration of the extract entirely protected against the unfavourable impact of Cd on these variables. Moreover, it is important to emphasize that for some of these parameters, the protective influence of the supplementation was evident only at some time points (Ca, Nrf2, KEAP1, and TIMP-4); at other time points, the supplementation did not provide any protection; or the effect was not observed at any time point (MMP-3). Apart from that, in the case of some parameters (Ca, Mg, TIMP-2, and TIMP-4) co-administration of the extract modified the Cd-alone unchanged values; however, such effects were noted rarely and occurred only in some time points.

Taking into account our previous findings in this experimental model and the properties of the AE ingredients, the beneficial impact of the extract supplementation against the Cd-induced changes in the values of various markers of neurotoxicity can be explained by its direct and indirect action. The first effect consists in the high antioxidative potential of the AE extract (reflected in its high ability to scavenge free radicals [36]) determined by its ingredients, including mainly polyphenols (especially anthocyanins), as well as carotenoids, vitamin E, and selenium [59,60,61,62]. Despite low bioavailability in the gastrointestinal tract and low penetration of the BBB, some polyphenols are known for their ability to cross this barrier and postpone the development of neurodegenerative diseases or improve their treatment [2]. The second main mechanism of the protective impact of the extract is related to the formation by polyphenols of stable complexes with ions of Cd (Cd^2+^) resulting in the diminished absorption of this element in the digestive tract and, thus, the lower body burden of Cd, including its lower accumulation in the brain and reducing its unfavourable action [33,34,35,36,37,38]. As with a moderate exposure, we have previously reported a lower Cd accumulation in the brain after 17 and 24 months, and in the brain mitochondria after 17 months, due to the AE supplementation; in the brain mitochondria after the 3-month low-level treatment [33,35], the protective impact of the extract administration might, at least to some extent, be related to lower Cd concentration in the nervous tissue. The beneficial properties of the AE can also be the effect of its other ingredients including vitamins C and E or tannins which constitute complexes with ions of metals [63,64], so that they can enhance the effects of polyphenolic compounds.

In our recent study [20], it was revealed that administration of the extract during the exposure to Cd at 1 mg/kg of diet entirely protected from a Cd-induced decrease in the value of TAS and an increase in TOS and, as a result, the development of oxidative stress and oxidative modifications of proteins, lipids, and DNA in the brain. The supplementation with the AE during the treatment with Cd at 5 mg/kg of diet also prevented the Cd destroying the oxidative–reductive balance and development of oxidative stress, except for only a partial protection against enhanced TOS and a lack of protection from an elevation of OSI after 3 months [20]. It is worth emphasising that the neuroprotective impact of AE, and the long-term moderate exposure to Cd, might be related to the entire protection of the extract against this heavy metal-induced decrease in the activities of antioxidative enzymes such as the SOD, CAT, and GPx already reported [20] in these animals. Excessive amounts of superoxide radicals and NO combined with impaired antioxidative enzymatic activity in the nervous cells are responsible for the generation of peroxynitrite, a dangerous reactive form of nitrogen. Because the neurotoxic action of Cd is connected to the pro-oxidative properties of this heavy metal, our study confirmed the relationship between the oxidative–antioxidative status of the nervous tissue and changes of the values of numerous biomarkers of neurotoxicity in the brain, and the protection from destroying the redox balance provided by the AE administration was allowed to weaken the unfavourable impact of this xenobiotic on the nervous tissue via oxidative stress. The numerous correlations noted between the investigated indices of neurotoxicity and the values of TOS, TAS, and OSI, as well as some dependencies with markers of oxidative modifications of cellular macromolecules (3-NT, PC, LPO, 8-iso, and γ-H2AX) in the brain of the animals administered with the AE, confirm an involvement of its strong antioxidative potential in the protection from Cd neurotoxicity.

Our research (presented in this paper and a recent report [20]) is the first that investigated and revealed the beneficial impact of the extract from *A. melanocarpa* berries against Cd-induced changes in the values of various markers of neurotoxicity. Moreover, one of the ingredients of AE, chlorogenic acid, can cross the BBB [65] and thus act directly in the brain complexing Cd^2+^, which can be a part of the direct mechanism of action of the extract. As discussed earlier in this paper, the supplementation with the AE modified the values of some Cd-alone unchanged parameters; however, only at some time points. Moreover, the 3- and 10-month administration of the extract alone increased the activity of Ca^2+^/Mg^2+^-ATPase but, at this stage of our research, we cannot explain these effects. 

Although other authors have not studied the impact of AE on Cd neurotoxicity, they have reported the protective efficacy of some AE ingredients against Cd-induced disturbances in the brain tissue [12,14,18,21,23,26]. The administration of ferulic acid (10 and 20 mg/kg b.w.) fully protected against a Cd-caused (2.5 mg/kg b.w., 21 days) increase in the activity of Na^+^/K^+^-ATPase in the brain, whereas chlorogenic acid (60 mg/kg b.w.) prevented a decrease in this enzyme activity due to exposure to Cd at a dose of 5 mg/kg b.w. for 30 days [12,18]. Moreover, chlorogenic acid (60 mg/kg b.w., administered under Cd treatment at the dose of 5 mg/kg b.w. for 30 days) and selenium (nanoparticles, 0.5 mg/kg b.w., under treatment with CdCl_2_ at a dose of 5 mg/kg b.w. twice a week for 8 weeks) prevented this heavy metal-caused decrease in the activity of AChE in this organ, while the administration of kaempferol (50 mg/kg b.w. under treatment with CdCl_2_ at a dose of 4.5 mg/kg b.w. for 30 days) served partial protection [14,18,21]. The effectiveness in the protection against changes in the concentration or expression of Nrf2 and KEAP1 was noted in the case of quercetin (25 mg/kg b.w., under Cd treatment at the dose of 5 mg/kg b.w. for 28 days) and selenium (0.5 mg/kg b.w., under Cd treatment at a dose of 1 mg/kg b.w. for 30 days) [23,26]. Quercetin was reported to provide partial protection against a Cd-induced decrease in the concentration of Nrf2 and an increase in the concentration of KEAP1 [26], whereas selenium provided protection against a rise in the expression of Nrf2 and a decrease in the expression of KEAP1 [23]. Taking into consideration the fact that, in the case of the beneficial impact of ferulic acid on the activity of Na^+^/K^+^-ATPase [12] and the effect of chlorogenic acid, selenium, and kaempferol on the activity of AChE [14,18,21], the direction of changes of these parameters in the brain was the same as in our study, it is possible that the impact of AE can be connected with the action of these ingredients. Moreover, evidence on the protective effect of other polyphenolic compounds, such as protocatechuic acid, curcumin, and gallic acid, against the damaging action of Cd in the nervous system was provided in the available literature [11,13,19,50].

We are aware, not only of the novelty and important practical implications of our research, but of its limitations. As this study was performed on female rats, the results can be interpreted only according to women. Because a wide range of parameters were assessed, the experiment included measurements of the whole brain, not its parts. Moreover, the animals examined after specific periods were not the same rats. This may explain why, in the case of some parameters, no change (or a tendency to change) was noted at some time points despite statistically significantly changed values of these parameters at other time points. The next limitation is a lack of determination of the concentrations of Ca^2+^, Mg^2+^, Na^+^, and K^+^; however, it was impossible because of the limited weight of the brain and the wide range of measurements. Thus, only total concentrations of Ca and Mg were assayed in the brain tissue. Moreover, at this stage of our study, we cannot explain the detailed mechanisms of Cd neurotoxicity and the protective impact of AE and show the extract ingredients responsible for this protection. However, our study provided unquestionable evidence that even low-level repeated exposure to Cd may create a risk of nervous system damage, while supplementation with aronia products may effectively counter this action. Despite the limitations of our research on the effect of low-level exposure to Cd on the nervous system and the possibility of protective use of AE, the results of this study have practical implications. They have shed new light on this heavy metal as a neurotoxic element, showed the possibility of using chokeberry products in preventing Cd neurotoxicity, and indicated directions for future research in this area. Aronia products seem to be an especially promising strategy for effective protection because, as was revealed in our studies [33,34,35,36,37,38], they may provide systemic protection against various effects of exposure to Cd. We are aware that further studies in this area are needed. The present study provided further evidence for the toxic action of Cd at a low exposure and a possible effective strategy for protection. Considering all our findings in this experimental model [20,33,34,35,36,37,38], it seems reasonable to evaluate the relationship between the consumption of aronia-based products and Cd concentrations, and the effects of its toxic action in the general population, before *A. melanocarpa* products might be recommended to use to counteract the unfavourable outcomes of environmental exposure to this heavy metal.

## 4. Materials and Methods

### 4.1. Chemicals and Reagents

Cadmium chloride was purchased from POCh (Gliwice, Poland) and butylhydroxytoluene was purchased from Sigma-Aldrich GmbH (Steinheim, Germany). Acetonitrile, as well as trace-pure concentrated acids such as nitric acid and hydrochloric acid, were received from Merck (Darmstadt, Germany). Stocks of standard Ca and Mg solutions assigned for the atomic absorption spectrometry (AAS) method were obtained from C.P.A. Ltd. (Stara Zagora, Bulgaria). The Standard Reference Material Bovine Liver (No. 1577b), used to check the analytical quality of these elements assay by the AAS method, was produced by the National Institute of Standards and Technology (Gaithersburg, MD, USA).

Commercial kits by Cloud-Clone Corp. (Katy, TX, USA) were used to assess the concentrations of AChE, CAM and NOS1. The activities of Ca^2+^/Mg^2+^-ATPase and Na^+^/K^+^-ATPase, as well as the concentrations of Nrf2 and KEAP1 were assayed with the use of kits provided by MyBioSource (San Diego, CA, USA). To assess the concentrations of MMPs and TIMPs kits, SunRed (Shanghai, China) was used; whereas, to determine the concentration of PLA2, the kit was provided by FineTest (Wuhan, China). Total protein concentration was determined using a commercial kit by Biomaxima (Lublin, Poland).

### 4.2. Cd Diet and the Extract from A. melanocarpa L. Berries

Cadmium chloride at the proper amount was added to the Labofeed B and Labofeed H diets (Label Food ‘Morawski’, Kcynia, Poland) at the production stage to obtain the diets containing Cd at the concentration of 1 or 5 mg/kg. The determination of Cd in the diets containing this element at the concentration of 1 and 5 mg/kg performed in our laboratory confirmed that its content was consistent with the values declared by the producer and reached 0.96–1.22 mg/kg and 4.39–5.45 mg/kg, respectively [33]. The standard Labofeed diet contained only trace amounts of Cd (0.0535–0.0633 mg/kg) [33].

The AE was prepared daily from the freeze-dried powdered extract by Adamed Consumer Healthcare (Tuszyn, Poland). According to the producer (Certificate KJ 4/2010; Butch No. M100703), it consisted of 65.74% of polyphenols and their main group was anthocyanins (18.65%). The analysis conducted by our research team revealed that the extract contained 61.24 ± 0.33% of total polyphenols (mean ± standard error), including 20.23 ± 0.13% of anthocyanins, 12.99 ± 0.11% of proanthocyanidins, 11.09 ± 0.09% of phenolic acids, 6.83 ± 0.01% of chlorogenic acid, and 2.19 ± 0.1% of flavonoids [34]. For the chokeberry extract, an Ultra Performance Liquid Chromatography (UPLC) polyphenolic profile has been reported elsewhere [34]. Moreover, according to the information provided by the producer, AE contained other substances such as pectins, carotenoids, sugar, sugar alcohols, triterpenes, phytosterols, minerals, and vitamins.

### 4.3. Animals and Design of the Study

**Figure 8:**
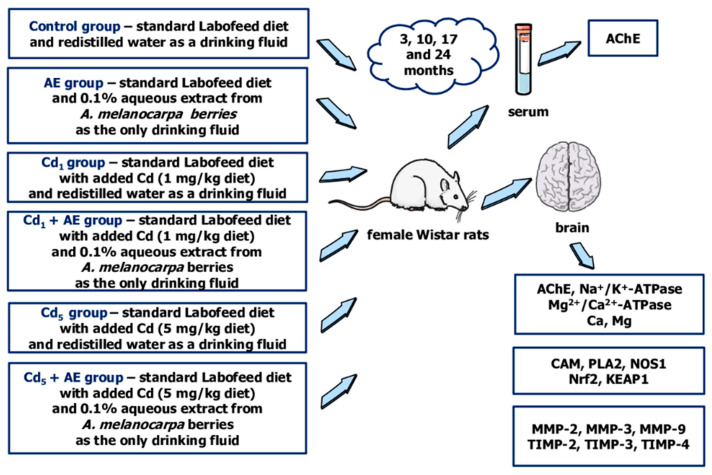
The schematic representation of the experimental model and measured parameters. AChE, acetylcholinesterase; AE, *Aronia melanocarpa* L. berries extract; Ca, calcium; Ca^2+^/Mg^2+^-ATPase, calcium-magnesium adenosine triphosphatase; CAM, calmodulin; KEAP1, Kelch-like ECH-associated protein 1; Mg, magnesium; MMP, metalloproteinase; Na^+^/K^+^-ATPase, sodium-potassium adenosine triphosphatase; NOS1, nitric oxide synthase 1; Nrf2, nuclear factor erythroid 2-related factor 2; PLA2, phospholipase A2; TIMP, tissue inhibitor of metalloproteinase. This figure was prepared using the templates available on the Servier Medical Art website (Creative Commons Attribution 4.0 unported license).

This study received approval from the Local Ethics Committee for Animal Experiments in Bialystok (Poland) (approval number 60/2009, 21 September 2009). It was performed on 192 female Wistar rats (3–4 weeks old) from Laboratory Animal House (Brwinów, Poland). Female animals were chosen for the experiment to investigate the impact of low-to-moderate exposure to Cd because they are known to be more sensitive to the toxic action of this xenobiotic than males [66]. The animals had 5 days of acclimatization to laboratory conditions. During the study period, the rats were kept in cages under standard conditions (12-h light-dark cycle, temperature 22 ± 2 °C, relative humidity 50 ± 10%) and they had ad libitum access to food (with and without Cd addition) and drinking fluids (redistilled water or AE). The animals were randomly divided into 6 experimental groups that received a diet containing Cd at the concentration of 0, 1, or 5 mg/kg diet (the control, Cd_1_, and Cd_5_ groups, respectively) and/or 0.1% AE as the only drinking fluid (the AE, Cd_1_ + AE, and Cd_5_ + AE groups) for 3, 10, 17, and 24 months. The redistilled water (drank by the groups that did not receive AE) contained Cd at a concentration < 0.05 μg/L. The schematic representation of the experimental model and measured parameters is shown in Figure 8.

The used levels of Cd dosage reflected lifetime low-level (1 mg/kg of diet) and moderate (5 mg/kg of diet) human exposure to this xenobiotic in developed countries [33]. Throughout the experiment, the mean daily intake of Cd was 39.2–83.8 µg/kg b.w., 37.5–84.9 µg/kg b.w., 210.1–403.2 µg/kg b.w., and 200.2–401.9 µg/kg b.w., respectively for the Cd_1_, Cd_1_ + AE, Cd_5_ and Cd_5_ + AE groups [33]. The concentration of Cd in the urine of animals was 0.085–0.285 and 0.284–0.695 μg/g creatinine in the Cd_1_ and Cd_5_ groups, respectively, whereas in the blood it reached 0.113–0.324 and 0.735–1.332 μg/L, respectively [33] and was comparable with the concentrations nowadays noted in the general population of different countries [5,6].

The intake of AE was within the range of 41.5–104.6 mg/kg b.w./24 h and did not differ between the experimental groups administered with the extract [33]. The daily consumption of polyphenols in the rats supplemented with the 0.1% AE was 2.9–7.3 times higher than their average intake in humans (1000 mg per day) [20,33,67].

There were no signs of morbidity during the whole experiment, but 3 animals died spontaneously between the 17th and 24th month (one from the AE, Cd_1_, and Cd_5_ groups). After 3, 10, 17, or 24 months the animals were sacrificed using Morbital (30 mg/kg b.w., administered intraperitoneally). The whole blood was taken (with and without anticoagulant) and different tissues and organs, including the brain, were dissected. The blood collected without anticoagulant was centrifuged after the coagulation and the serum was separated. The brain was frozen immediately and was stored at −70 °C before performing all planned analyses. The experimental model has been described by us in detail previously [20,33,34,35,36,37,38].

### 4.4. Determination of Biomarkers of Neurotoxicity in the Brain

The brain was sectioned longitudinally into two halves (comprising all structures of this organ). One of them was used to prepare (with the use of the Schütt Homogenplus homogenizer; Labortechnik GmbH, Göttingen, Germany) 20% (weight/volume) homogenates of the brain tissue in a potassium phosphate buffer (pH = 7.4) with the addition of butylhydroxytoluene in acetonitrile as previously reported [20]. The aliquots obtained by the centrifugation of the homogenates (3000× *g*, 10 min, at 4 °C) were kept frozen (−70 °C) until all parameters were assayed. To perform particular measurements in the aliquots, commercial enzyme-linked immunosorbent assay (ELISA) kits were used (Table 5). The assays were performed strictly according to the recommendations of the kit producers’ (Table 5) and the usage of the spectrophotometers MULTISCAN GO (Thermo Scientific, Vantaa, Finland). To express the precision of measurements of determined variables, intra- and inter-assay coefficients of variation (intra-CV and inter-CV, respectively) were estimated. The intra-CV was from <0.3% to <8.3%, whereas the inter-CV was from <0.3% to <8.8%, depending on the parameter (Table 5).

The concentrations were measured for all assayed variables except for Na^+^/K^+^-ATPase and Ca^2+^/Mg^2+^-ATPase for which the activities in the brain tissue were determined. The values of all variables were adjusted to the concentration of total protein in the brain assayed (CV < 3%) using a BioMaxima kit (No. 1-055-0200).

### 4.5. Determination of Ca and Mg in the Brain

The second part of the brain was subjected to wet digestion with a mixture of concentrated nitric acid and hydrochloric acid as described in the previous study [33]. Slices of the brain of the known weight were subjected to the process of wet digestion using a 9:1 mixture of trace-pure concentrated acids—nitric acid (65% HNO_3_) and hydrochloric acid (30% HCl) in a microwave system (Multiwave, Anton Paar GmbH, Graz, Austria). An excessive amount of acid was then evaporated by warming, after which the samples were diluted using ultra-pure water up to 10 mL.

The concentrations of Ca and Mg in such preparations (after appropriate dilution if necessary) were determined by flame (air–acetylene burner atomization) AAS method with the use of the atomic absorption spectrophotometer Z-5000 by Hitachi (Tokyo, Japan) and stocks of standard solutions of these elements assigned for the AAS method and the resonance line of 422.7 nm for Ca and 285.2 nm for Mg. The concentrations of Ca and Mg in the Standard Reference Material Bovine Liver (No. 1577b) were 116 ± 4 μg/g and 601 ± 28 μg/g, respectively, and the noticed values were 110.8 ± 3.8 μg/g and 582 ± 17 μg/g, respectively. The recovery of the metals in the reference material was 95.5% and 97% for Ca and Mg, respectively, and the CV was <3.4% and <2.9%, respectively. A quality assessment of these elements assay, based on the analysis of reference material, the recovery, and the CV, confirmed the reliability of the measurements.

### 4.6. Determination of AChE in the Serum

The determination of the activity of AChE in the serum was performed by colorimetric Ellman’s method (CV < 3.5%) [68]. The assay is based on the measurement of the rate of the production of thiocholine (a result of acetylcholine hydrolysis) in the continuous reaction of the thiol compound with 5,5′-dithiobis-2-nitrobenzoic acid. In this reaction, a yellow anion of 5-thio-2-nitro-benzoic acid is formed and the intensity of the production of the colour is assayed spectrophotometrically at 412 nm [68].

### 4.7. Statistical Analysis

The Statistica 13.3 package (StatSoft, Tulsa, OK, USA) was used to perform the statistical analysis of the results. To verify the normality of data distribution a Shapiro–Wilk test was performed. As no normal distribution of data was found in all experimental groups, to determine the statistical significance (*p* < 0.05) of differences between the groups a Kruskall–Wallis test was conducted. For all the measured parameters, median, minimum and maximum values, and 25–75% confidence interval are shown in figures (for details regarding the numerical values, please refer to the Supplementary Materials). Moreover, quantitative differences between the Cd_1_, Cd_5_, Cd_1_ + AE, and Cd_5_ + AE groups and the control group, as well as between the Cd_1_ + AE and Cd_5_ + AE groups and the appropriate Cd group (the Cd_1_ group and Cd_5_ group, respectively) are provided in figures (marked as a percentage difference or a factor of difference if the values were more than 100% higher or more than 100% lower). Moreover, the effect size demonstrated as eta squared (η^2^) and defined as the strength of the difference between the tested groups at each time point was calculated (large for η^2^ ≥ 0.14, medium for η^2^ between 0.01 and 0.14, and weak for η^2^ ≤ 0.01).

To predict the mutual dependencies between the measured variables, as well as the dependencies between these parameters and the concentration of Cd in the blood, urine, and brain (published previously in [33]), as well as the indices of the oxidative–reductive balance (OSI, TAS, and TOS) and markers of oxidative modifications of proteins, lipids, and DNA (3-NT, PC, LPO, 8-iso, and γ-H2AX) (published previously in [20]) in the brain, a Spearman’s correlation test was performed to evaluate an occurrence of a relationship between two variables and the degree of the correlation (r) (weak for |r| = 0.2–0.4, moderate for |r| = 0.4–0.7, strong for |r| = 0.7–0.9, and very strong for |r| > 0.9).

## 5. Conclusions

Based on the results of the current study it can be concluded that Cd at low-level and moderate exposure provokes neurotoxic effects by influencing proteins regulating redox homeostasis, neurotransmission, ions homeostasis, cell differentiation, and BBB functions. Moreover, the simultaneous administration of an extract from the berries of *A. melanocarpa* alleviated these toxic actions. Summing up, chokeberry products can be a potential preventive strategy against Cd-induced neurotoxicity at low-to-moderate exposure to this xenobiotic. The findings show that more attention must be paid to Cd as an environmental risk factor for nervous system damage and that consumption of widely available—and known to have numerous health benefits—natural products such as the chokeberry may facilitate to counteract the effect of this toxic heavy metal. Further research should be undertaken to completely investigate the mechanisms of the protective action of *A. melanocarpa* berries and to establish the effective amount of this natural product so that in the future it can be the potential preventive factor in the population exposed to Cd.

## Figures and Tables

**Table 1:** Relationships between the parameters measured in the present study in the aliquots of the brain homogenates and previously reported cadmium (Cd) concentration in the brain, blood, and urine in female rats that were or were not supplemented with 0.1% aqueous extract from *Aronia melanocarpa* L. berries (AE).

Parameter	Cd in the Brain ^1^		Cd in the Blood		Cd in the Urine	
	Without AE ^2^	With AE ^3^	Without AE	With AE	Without AE	With AE
AChE	−0.309 ^† 4^	0.279 ^†^	−0.397 ^‡^	NS	−0.250 *	NS
Na^+^/K^+^-ATPase	0.280 ^†^	NS	NS	NS	NS	NS
Ca^2+^/Mg^2+^-ATPase	NS	NS	NS	NS	NS	NS
Ca	NS	NS	0.387 ^‡^	NS	0.263 *	NS
Mg	0.260 *	NS	NS	NS	NS	NS
CAM	0.296 ^‡^	−0.308 ^†^	0.472 ^‡^	−0.464 ^‡^	0.409 ^‡^	−0.469 ^‡^
PLA2	NS	NS	NS	NS	NS	NS
NOS1	NS	0.263 *	NS	NS	NS	NS
Nrf2	−0.432 ^‡^	NS	−0.567 ^‡^	NS	−0.452 ^‡^	NS
KEAP1	−0.501 ^‡^	NS	−0.561 ^‡^	NS	−0.490 ^‡^	NS
MMP-2	NS	NS	NS	0.264 *	NS	0.243 *
MMP-3	NS	NS	NS	NS	NS	NS
MMP-9	0.451 ^‡^	−0.228 *	0.572 ^‡^	−0.360 ^‡^	0.601 ^‡^	−0.344 ^‡^
TIMP-2	NS	NS	NS	NS	NS	NS
TIMP-3	NS	−0.372 ^‡^	NS	NS	NS	−0.220 *
TIMP-4	−0.318 ^†^	0.270 ^†^	−0.246 *	0.553 ^‡^	−0.302 ^†^	0.468 ^‡^

^1^ The concentration of Cd in the brain, blood, and urine was provided in the previous publication of our research team [33]. ^2^ The animals that were not supplemented with AE (the control group, Cd_1_ group, and Cd_5_ group). ^3^ The animals that received supplementation with AE (the AE group, Cd_1_ + AE group, and Cd_5_ + AE group). ^4^ The data show the results of Spearman’s correlation analysis presented as a correlation coefficient (r) and the level of statistical significance (* *p* < 0.05, ^†^ *p* < 0.01, and ^‡^ *p* < 0.001), or NS for *p* > 0.05 (lack of relationship). AChE, acetylcholinesterase; Ca, calcium; Ca^2+^/Mg^2+^-ATPase, calcium-magnesium adenosine triphosphatase; CAM, calmodulin; KEAP1, Kelch-like ECH-associated protein 1; Mg, magnesium; MMP, metalloproteinase; Na^+^/K^+^-ATPase, sodium-potassium adenosine triphosphatase; NOS1, nitric oxide synthase 1; Nrf2, nuclear factor erythroid 2-related factor 2; PLA2, phospholipase A2; TIMP, tissue inhibitor of metalloproteinase.

**Table 2:** Relationships between the parameters measured in the present study and previously reported total oxidative status (TOS), total antioxidative status (TAS), and oxidative stress index (OSI) in the aliquots of the brain homogenates of female rats supplemented or not with 0.1% aqueous extract from *Aronia melanocarpa* L. berries (AE).

Parameter	TOS ^1^		TAS		OSI	
	Without AE ^2^	With AE ^3^	Without AE	With AE	Without AE	With AE
AChE	−0.276 * ^4^	NS	0.391 ^‡^	NS	−0.536 ^‡^	NS
Na^+^/K^+^-ATPase	NS	NS	−0.427 ^‡^	NS	0.279 ^†^	NS
Ca^2+^/Mg^2+^-ATPase	NS	NS	NS	0.244 *	NS	−0.214 *
Ca	NS	−0.275 ^†^	−0.732 ^‡^	−0.437 ^‡^	0.528 ^‡^	NS
Mg	NS	0.264 *	0.259 *	0.206 *	NS	NS
CAM	0.311 ^†^	NS	NS	NS	0.472 ^†^	NS
PLA2	NS	NS	0.566 ^‡^	−0.245 *	−0.219 *	NS
NOS1	NS	NS	NS	0.311 ^†^	NS	NS
Nrf2	−0.479 ^‡^	0.365 ^‡^	0.687 ^‡^	0.579 ^‡^	−0.700 ^‡^	NS
KEAP1	−0.546 ^‡^	NS	0.606 ^‡^	0.371 ^‡^	−0.659 ^‡^	NS
MMP-2	NS	0.257 *	0.532 ^‡^	NS	−0.281 ^†^	NS
MMP-3	NS	0.279 ^†^	NS	0.212 *	NS	NS
MMP-9	0.479 ^‡^	NS	NS	0.296 ^†^	0.363 ^‡^	NS
TIMP-2	NS	NS	0.416 ^‡^	NS	NS	NS
TIMP-3	NS	−0.347 ^‡^	−0.720 ^‡^	−0.382 ^‡^	0.250 *	NS
TIMP-4	−0.223 *	NS	NS	−0.249 *	−0.226 *	0.211 *

^1^ The values of TOS, TAS, and OSI were provided in the previous publication of our research team [20]. ^2^ The animals that were not supplemented with AE (the control group, Cd_1_ group, and Cd_5_ group). ^3^ The animals that received supplementation with AE (the AE group, Cd_1_ + AE group, and Cd_5_ + AE group). ^4^ The data show the results of a Spearman’s correlation analysis presented as a correlation coefficient (r) and the level of statistical significance (* *p* < 0.05, ^†^ *p* < 0.01, and ^‡^ *p* < 0.001), or NS for *p* > 0.05 (lack of relationship). AChE, acetylcholinesterase; Ca, calcium; Ca^2+^/Mg^2+^-ATPase, calcium-magnesium adenosine triphosphatase; CAM, calmodulin; KEAP1, Kelch-like ECH-associated protein 1; Mg, magnesium; MMP, metalloproteinase; Na^+^/K^+^-ATPase, sodium-potassium adenosine triphosphatase; NOS1, nitric oxide synthase 1; Nrf2, nuclear factor erythroid 2-related factor 2; PLA2, phospholipase A2; TIMP, tissue inhibitor of metalloproteinase.

**Table 3:** Relationships between the parameters measured in the present study and previously reported biomarkers of oxidative damage to proteins, lipids and deoxyribonucleic acid (DNA) in the aliquots of the brain homogenates of female rats supplemented or not with 0.1% aqueous extract from *Aronia melanocarpa* L. berries (AE).

Parameter	3-NT ^1^		PC		LPO		8-iso		γ-H2AX	
	Without AE ^2^	With AE ^3^	Without AE	With AE	Without AE	With AE	Without AE	With AE	Without AE	With AE
AChE	−0.215 * ^4^	NS	−0.325 ^†^	NS	−0.292 ^†^	NS	−0.396 ^‡^	NS	−0.246 *	NS
Na^+^/K^+^-ATPase	NS	NS	NS	NS	NS	0.218 *	NS	NS	−0.273 *	NS
Ca^2+^/Mg^2+^-ATPase	NS	NS	NS	0.242 *	NS	0.339 ^‡^	NS	NS	0.238 *	NS
Ca	NS	−0.304 ^†^	0.470 ^‡^	NS	NS	−0.263 *	0.591 ^‡^	NS	0.391 ^‡^	−0.263 *
Mg	NS	0.210 *	NS	−0.254 *	NS	NS	NS	NS	NS	NS
CAM	0.411 ^‡^	0.235 *	0.459 ^‡^	−0.222 *	0.371 ^‡^	NS	0.647 ^‡^	NS	0.415 ^‡^	NS
PLA2	NS	NS	NS	NS	NS	NS	−0.324 ^†^	NS	NS	NS
NOS1	0.256 *	NS	0.416 ^‡^	NS	0.241 *	0.239 *	0.318 ^†^	NS	0.522 ^‡^	0.287 ^†^
Nrf2	NS	0.613 ^‡^	−0.495 ^‡^	NS	−0.334 ^‡^	0.508 ^‡^	−0.622 ^‡^	NS	−0.284 ^†^	0.375 ^‡^
KEAP1	NS	0.456 ^‡^	−0.471 ^‡^	NS	−0.317 ^†^	0.497 ^‡^	−0.467 ^‡^	NS	−0.299 ^†^	0.407 *
MMP-2	NS	NS	−0.278 ^†^	NS	NS	0.324 ^†^	−0.254 *	NS	NS	NS
MMP-3	NS	NS	NS	NS	NS	0.209 *	NS	NS	NS	NS
MMP-9	0.393 ^‡^	0.324 ^†^	0.485 ^‡^	NS	0.528 ^‡^	NS	0.206 *	NS	0.246 *	NS
TIMP-2	NS	NS	NS	0.386 ^‡^	NS	NS	NS	−0.221 *	NS	NS
TIMP-3	−0.208 *	NS	0.256 *	NS	NS	NS	0.501 ^‡^	0.343 ^‡^	0.315 ^†^	NS
TIMP-4	NS	−0.360 ^‡^	NS	0.229 *	NS	NS	NS	−0.371 ^‡^	0.209 *	−0.238 *

^1^ The values of 3-NT, PC, LPO, 8-iso, and γ-H2AX were provided in the previous publication of our research team [20]. ^2^ The animals that were not supplemented with AE (the control group, Cd_1_ group, and Cd_5_ group). ^3^ The animals that received supplementation with AE (the AE group, Cd_1_ + AE group, and Cd_5_ + AE group). ^4^ The data show the results of a Spearman’s correlation analysis presented as a correlation coefficient (r) and the level of statistical significance (* *p* < 0.05, ^†^ *p* < 0.01, and ^‡^ *p* < 0.001), or NS for *p* > 0.05 (lack of relationship). AChE, acetylcholinesterase; Ca, calcium; Ca^2+^/Mg^2+^-ATPase, calcium-magnesium adenosine triphosphatase; CAM, calmodulin; KEAP1, Kelch-like ECH-associated protein 1; LPO, lipid peroxides; Mg, magnesium; MMP, metalloproteinase; Na^+^/K^+^-ATPase, sodium-potassium adenosine triphosphatase; NOS1, nitric oxide synthase 1; Nrf2, nuclear factor erythroid 2-related factor 2; PLA2, phospholipase A2; PC, protein carbonyl groups; TIMP, tissue inhibitor of metalloproteinase; γ-H2AX, γ-H2A histone family member; 3-NT, 3-nitrotyrosine; 8-iso, 8-isoprostane.

**Table 4:** Relationships between the parameters determined in the aliquots of the brain homogenates of rats administered (*italics*) or not with 0.1% aqueous extract from *Aronia melanocarpa* L. berries (AE).

Parameter	AChE	Na^+^/K^+^ -ATPase	Ca^2+^/Mg^2+^-ATPase	Ca	Mg	CAM	PLA2	NOS1	Nrf2	KEAP1	MMP-2	MMP-3	MMP-9	TIMP-2	TIMP-3	TIMP-4
AChE	-	*NS*	*NS*	*NS*	*NS*	*NS*	*NS*	*NS*	*NS*	*NS*	*NS*	*NS*	*NS*	*NS*	*NS*	*NS*
Na^+^/K^+^-ATPase	NS ^1^	-	*0.503* ^‡^	*NS*	NS	*0.272* ^†^	*−0.246* *	*NS*	*NS*	*NS*	*0.641* ^‡^	*0.309* ^†^	*0.517* ^‡^	*0.578* ^‡^	*NS*	*0.390* ^‡^
Ca^2+^/Mg^2+^-ATPase	NS	−0.461 ^‡^	-	*NS*	*NS*	*NS*	*NS*	*0.277* ^†^	*0.215* *	*NS*	*0.318* ^†^	*NS*	*0.398* ^‡^	*0.552* ^‡^	*−0.252* *	*0.249* *
Ca	−0.344 ^†^	NS	NS	-	*−0.215* *	*NS*	*NS*	*NS*	*−0.384* ^‡^	*−0.390* ^‡^	*NS*	*NS*	*NS*	*NS*	*0.423* ^‡^	*0.254* *
Mg	NS	0.214 *	−0.419 ^‡^	NS	-	*NS*	*NS*	*NS*	*NS*	*0.236* *	*NS*	*NS*	*0.207* *	*−0.284* ^†^	*NS*	*−0.324* ^†^
CAM	−0.457 ^‡^	NS	NS	0.350 ^‡^	NS	-	*NS*	*NS*	*NS*	*NS*	*0.215* *	*NS*	*0.416* ^‡^	*NS*	*NS*	*NS*
PLA2	NS	NS	NS	−0.437 ^‡^	0.332 ^†^	NS	-	*NS*	*−0.255* *	*NS*	*−0.292* ^‡^	*NS*	*NS*	*NS*	*0.248* *	*NS*
NOS1	NS	−0.438 ^‡^	NS	NS	−0.267 ^†^	0.271 *	NS	-	*0.361* ^‡^	*NS*	*NS*	*NS*	*NS*	*NS*	*−0.240* *	*NS*
Nrf2	0.595 ^‡^	−0.509 ^‡^	NS	−0.601 ^‡^	NS	−0.378 ^‡^	0.437 ^‡^	NS	-	*0.469* ^‡^	*0.225* *	*NS*	*0.329* ^†^	*NS*	*−0.413* ^‡^	*−0.231* *
KEAP1	0.554 ^‡^	−0.383 ^‡^	NS	NS	NS	−0.274 *	0.329 ^†^	NS	0.437 ^‡^	-	*NS*	*NS*	*NS*	*NS*	*−0.283* ^†^	*−0.313* ^†^
MMP-2	0.335 ^†^	−0.533 ^‡^	NS	−0.304 ^†^	NS	NS	0.346 ^‡^	NS	0.498 ^‡^	0.448 ^‡^	-	*0.440* ^‡^	*0.472* ^‡^	*0.549* ^‡^	*NS*	*0.443* ^‡^
MMP-3	NS	−0.352 ^‡^	NS	NS	NS	NS	NS	0.203 *	NS	NS	NS	-	*0.209* *	*NS*	*NS*	*NS*
MMP-9	NS	NS	NS	0.213 *	NS	0.387 ^‡^	0.249 *	0.241 *	NS	−0.209 *	NS	NS	-	*0.380* ^‡^	*NS*	*NS*
TIMP-2	NS	−0.581 ^‡^	0.347 ^‡^	NS	NS	NS	0.316 ^†^	NS	0.383 ^‡^	0.388 ^‡^	0.493 ^‡^	NS	0.352 ^‡^	-	*NS*	*0.568* ^‡^
TIMP-3	NS	NS	NS	0.611 ^‡^	−0.294 ^†^	NS	−0.408 ^‡^	NS	−0.398 ^‡^	−0.315 ^†^	−0.207 *	NS	NS	NS	-	*NS*
TIMP-4	NS	−0.519 ^‡^	0.399 ^‡^	NS	NS	NS	NS	0.260 *	0.208 *	0.249 *	NS	NS	NS	0.359 ^‡^	NS	-

^1^ The data show the results of a Spearman’s correlation analysis presented as a correlation coefficient (r) and the level of statistical significance (* *p* < 0.05, ^†^ *p* < 0.01, and ^‡^ *p* < 0.001), and NS for *p* > 0.05 (lack of relationship). The values in *italics* refer to the animals supplemented with AE (the AE group, Cd_1_ + AE group, and Cd_5_ + AE group). Other values refer to the animals that did not receive supplementation with AE (the control group, Cd_1_ group, and Cd_5_ group). AChE, acetylcholinesterase; AE, *Aronia melanocarpa* L. berries extract; Ca, calcium; Ca^2+^/Mg^2+^-ATPase, calcium-magnesium adenosine triphosphatase; CAM, calmodulin; KEAP1, Kelch-like ECH-associated protein 1; Mg, manganese; MMP, metalloproteinase; Na^+^/K^+^-ATPase, sodium-potassium adenosine triphosphatase; NOS1, nitric oxide synthase 1; Nrf2, nuclear factor erythroid 2-related factor 2; PLA2, phospholipase A2; TIMP, tissue inhibitor of metalloproteinase.

**Table 5:** Data on the commercial kits used for determination of particular parameters together with the intra-assay coefficients of variation (intra-CV) and inter-assay coefficients of variation (inter-CV) for these measurements.

Parameter	Kind of Commercial Kit	Precision of Measurements	
		Intra-CV	Inter-CV
AChE	ELISA Kit for Acetylcholinesterase (AChE) (No. SEB447Ra) Cloud-Clone Corp.	<5.3% and <6.6%	<4.2%
Ca^2+^/Mg^2+^-ATPase	Rat Ca-Mg-ATPase ELISA Kit (No. MBS3809417) MyBiosource	<0.3% and <2%	<0.3%
Na^+^/K^+^-ATPase	Rat Na-K-ATPase ELISA Kit (No. MBS3809783) MyBiosource	<2.2% and <4%	<3%
CAM	ELISA Kit for Calmodulin (CAM) (No. SEA640Ra) Cloud-Clone Corp.	<4% and <5%	<6%
PLA2	Rat PLA2G2A (Phospholipase A2, Group IIA) ELISA Kit (No. ER1276), FineTest	<7.1% and <8.3%	<5.2%
NOS1	ELISA Kit for Nitric Oxide Synthase 1, Neuronal (NOS1) (No. SEA815Ra), Cloud-Clone Corp.	<6.6% and <5%	<2.5%
Nrf2	Rat Nuclear Factor Erythroid 2-Related Factor 2 (NRF2) ELISA Kit (No. MBS012148), MyBiosource	<3.6% and <7.9%	<3.2%
KEAP1	Rat Kelch like ECH Associated Protein 1 (KEAP1) ELISA kit (No. MBS7218529), MyBiosource	<3% and <8%	<3.3%
MMP-2	Rat (MMP-2) ELISA Kit (No. 201-11-0136) SunRed	<4% for both kits	<5.4%
MMP-3	Rat (MMP-3) ELISA Kit (No. 201-11-0317) SunRed	<3% and <4%	<4%
MMP-9	Rat (MMP-9) ELISA Kit (No. 201-11-0322) SunRed	<4.6% and <3.7%	<8.8%
TIMP-2	Rat (TIMP-2) ELISA Kit (No. 201-11-0324) SunRed	<2.5% and <2.3%	<2.8%
TIMP-3	Rat (TIMP-3) ELISA Kit (No. 201-11-0325) SunRed	<2% and <1%	<2%
TIMP-4	Rat (TIMP-4) ELISA Kit (No. 201-11-0326) SunRed	<3% and <1%	<1%

AChE, acetylcholinesterase; Ca^2+^/Mg^2+^-ATPase, calcium-magnesium adenosine triphosphatase; CAM, calmodulin; KEAP1, Kelch-like ECH-associated protein 1; MMP, metalloproteinase; Na^+^/K^+^-ATPase, sodium-potassium adenosine triphosphatase; NOS1, nitric oxide synthase 1; Nrf2, nuclear factor erythroid 2-related factor 2; PLA2, phospholipase A2; TIMP, tissue inhibitor of metalloproteinase.

## Data Availability

The original contributions presented in the study are included in the article/Supplementary Materials, further inquiries can be directed to the corresponding author.

## Supplementary Materials

The supporting information can be downloaded at: https://www.mdpi.com/article/10.3390/ijms252010887/s1.

## Abbreviations

AChE, acetylcholinesterase; AE, *Aronia melanocarpa* L. berries extract; b.w., body weight; BBB, blood-brain barrier; Ca, calcium; Ca^2+^, calcium ion; Ca^2+^/Mg^2+^-ATPase, calcium-magnesium adenosine triphosphatase; CAM, calmodulin; CAT, catalase; Cd, cadmium; c-GMP, cyclic guanosine 3′,5′-monophosphate; DNA, deoxyribonucleic acid; CV, coefficient of variation; ELISA, enzyme-linked immunosorbent assay; intra-CV, intra-assay coefficient of variation; inter-CV, inter-assay coefficient of variation; GPx, glutathione peroxidase; KEAP1, Kelch-like ECH-associated protein 1; K^+^, potassium ion; LPO, lipid peroxides; Mg, magnesium; MMP, metalloproteinase; Na^+^, sodium ion; Na^+^/K^+^-ATPase, sodium-potassium adenosine triphosphatase; NO, nitric oxide; NOS1, nitric oxide synthase 1; Nrf2, nuclear factor erythroid 2-related factor 2; OSI, oxidative stress index; PC, protein carbonyl groups; PLA2, phospholipase A2; SOD, superoxide dismutase; TAS, total antioxidative status; TIMP, tissue inhibitor of metalloproteinase; TOS, total oxidative status; γ-H2AX, γ-H2A histone family member X; 3-NT, 3-nitrotyrosine; 8-iso, 8-isoprostane.
