# Supplementary material for: The Protective Effect of the Supplementation with an Extract from Aronia melanocarpa L. Berries against Cadmium-Induced Changes of Chosen Biomarkers of Neurotoxicity in the Brain—A Study in a Rat Model of Current Lifetime Human Exposure to This Toxic Heavy Metal
Source: Int J Mol Sci. 2024 Oct 10;25(20):10887. doi: 10.3390/ijms252010887 (PMC11507053; doi:10.3390/ijms252010887)
Supplement: Supplementary file 1 [file ijms-25-10887-s001.zip › ijms-3174684-supplementary.pdf]

International Journal of *Molecular Sciences*

Supplementary Material

The Protective Effect of the Supplementation with an Extract from *Aronia melanocarpa* L. Berries against Cadmium-Induced Changes of Chosen Biomarkers of Neurotoxicity in the Brain—A Study in a Rat Model of Current Lifetime Human Exposure to This Toxic Heavy Metal

Agnieszka Ruczaj, Joanna Rogalska, Małgorzata Gałażyn-Sidorczuk and Małgorzata M. Brzóska *

Department of Toxicology, Medical University of Bialystok, Adama Mickiewicza 2C Street, 15-222 Bialystok, Poland; agnieszka.ruczaj@sd.umb.edu.pl (A.R.); joanna.rogalska@umb.edu.pl (J.R.); malgorzata.galazyn-sidorczuk@umb.edu.pl (M.G.-S.)

* Correspondence: malgorzata.brzoska@umb.edu.pl; Tel.: +48-85-748-5604; Fax: +48-85-748-5834

Citation: Ruczaj, A.; Rogalska, J.; Gałażyn-Sidorczuk, M.; Brzóska, M.M. The Protective Effect of the Supplementation with an Extract from *Aronia melanocarpa* L. Berries against Cadmium-Induced Changes of Chosen Biomarkers of Neurotoxicity in the Brain—A Study in a Rat Model of Current Lifetime Human Exposure to This Toxic Heavy Metal. *Int. J. Mol. Sci.* **2024**, *25*, 10887. https://doi.org/10.3390/ijms252010887

Academic Editors: Manuel Aureliano, Juan Llopis and Agnieszka Scibior

Received: 9 August 2024
Revised: 2 October 2024
Accepted: 7 October 2024
Published: 10 October 2024

Copyright: © 2024 by the authors. Licensee MDPI, Basel, Switzerland. This article is an open access article distributed under the terms and conditions of the Creative Commons Attribution (CC BY) license (https://creativecommons.org/licenses/by/4.0/).

Table S1. The concentration of acetylcholinesterase (AChE) in the aliquots of the brain homogenates and its activity in the serum of female rats.

Group	Experiment Duration			
	**3 Months**	**10 Months**	**17 Months**	**24 Months**
**AChE in the Brain (ng/mg protein)**				
Control	2.642<br>2.230–4.185	2.298<br>1.867–6.756	3.896<br>1.583–5.368	3.070<br>2.177–6.516
AE	2.335<br>1.998–4.906	2.983<br>1.244–7.199	2.532<br>1.923–4.768	3.188<br>1.459–6.377
Cd_1	1.963<br>1.089–3.660	1.997<br>1.579–3.915	1.410 ^{a*}<br>0.835–1.995	1.030 $^{a\dagger\, b\dagger}$<br>0.935–1.269
Cd_1 + AE	4.070 ^{c*}<br>2.817–5.987	2.560<br>2.036–6.376	2.604<br>1.302–6.376	3.572 ^{c†}<br>1.208–5.263
Cd_5	1.989 ^{d*}<br>1.118–3.741	2.302<br>1.845–6.485	1.778<br>0.682–2.864	1.307<br>0.934–1.850
Cd_5 + AE	3.426<br>1.676–5.782	4.377<br>1.364–6.501	4.667 ^{c†}<br>1.465–6.179	2.765 ^{c*}<br>1.914–5.717
Effect size (η^2)	0.241	-	0.391	0.555
**AChE in the Serum (U/L)**				
Control	7.574<br>4.338–11.03	7.485<br>3.417–11.51	10.00<br>4.485–14.12	11.96<br>6.618–20.66
AE	9.439<br>4.706–18.97	14.10<br>10.93–19.88	9.614<br>6.029–11.03	14.03<br>8.824–17.35
Cd_1	7.132<br>2.794–13.60	16.10 ^{a†}<br>9.142–20.66	20.12 ^{b*}<br>10.00–32.28	9.905<br>4.044–13.97
Cd_1 + AE	10.54<br>4.485–30.81	14.28<br>10.96–19.46	10.24<br>6.324–12.79	10.63<br>6.618–14.78
Cd_5	7.188<br>3.897–8.971	15.84 ^{a†}<br>7.672–23.50	24.98 $^{a\dagger\, b*\, d\dagger}$<br>11.03–36.03	8.634<br>5.809–10.96
Cd_5 + AE	6.673<br>3.750–8.309	10.74<br>6.887–16.30	13.04<br>8.750–16.69	10.51<br>6.103–17.57
Effect size (η^2)	-	0.349	0.505	-

Cadmium (Cd) (0, 1, or 5 mg Cd/kg diet for the Control, Cd_1, and Cd_5 groups respectively) and/or extract from *Aronia melanocarpa* L. berries (AE, Cd_1 + AE, and Cd_5 + AE groups) were administered to rats for 3, 10, 17, and 24 months. The presented data are median, minimum and maximum values for rats in each group (8 animals, except for 7 females in the AE, Cd_1, and Cd_5 groups after 24 months). The statistically significant differences are marked as * $p < 0.05$, and $^{\dagger} p < 0.01$ compared to: a – Control group, b – AE group, c – Cd_1 group, and d – Cd_1 + AE group.

Table S2. The activities of sodium-potassium adenosine triphosphatase (Na^+/K^+-ATPase) and calcium-magnesium adenosine triphosphatase (Ca^{2+}/Mg^{2+}-ATPase) in the aliquots of the brain homogenates of female rats.

Group	Experiment Duration			
	3 Months	10 Months	17 Months	24 Months
**Na^+/K^+-ATPase (U/mg protein)**				
Control	0.378<br>0.120–0.517	0.729<br>0.308–1.136	0.490<br>0.233–0.706	0.481<br>0.241–0.563
AE	0.309<br>0.057–0.588	0.620<br>0.224–1.070	0.422<br>0.142–0.536	0.540<br>0.150–0.832
Cd_1	0.541<br>0.368–0.869	0.655<br>0.340–0.940	0.476<br>0.247–0.645	0.526<br>0.376–0.732
Cd_1 + AE	0.581<br>0.096–0.801	0.776<br>0.188–1.154	0.508<br>0.220–1.271	0.652<br>0.229–0.967
Cd_5	0.384<br>0.219–0.620	0.703<br>0.415–1.103	1.076 $^{a* \, b† \, c*}$<br>0.594–1.275	0.766 $^{a† \, b*}$<br>0.704–1.008
Cd_5 + AE	0.376<br>0.212–0.419	0.591<br>0.365–0.626	0.469 ^{e*}<br>0.373–0.565	0.519 ^{e*}<br>0.468–0.589
Effect size (η2)	-	-	0.312	0.360
**Ca^{2+}/Mg^{2+}-ATPase (mU/mg protein)**				
Control	11.19<br>8.457–20.67	6.725<br>5.112–11.30	16.85<br>11.38–20.48	17.44<br>9.467–23.52
AE	23.09 ^{a*}<br>12.35–67.00	12.45 ^{a†}<br>8.931–16.76	17.02<br>13.40–18.91	13.84<br>6.801–27.33
Cd_1	16.38<br>12.24–21.44	11.32<br>7.147–26.55	14.61<br>11.49–19.37	15.38<br>8.399–21.85
Cd_1 + AE	20.88<br>10.18–36.06	6.921 ^{b*}<br>4.097–10.88	11.43 ^{b*}<br>8.217–16.45	16.35<br>6.519–21.98
Cd_5	16.09<br>4.045–31.67	8.579<br>6.302–10.61	13.57<br>5.551–16.01	12.81<br>11.01–24.80
Cd_5 + AE	23.29<br>12.63–28.90	9.374<br>6.569–12.45	11.12<br>7.482–18.67	16.66<br>11.23–26.54
Effect size (η^2)	0.179	0.334	0.271	-

Cadmium (Cd) (0, 1, or 5 mg Cd/kg diet for the Control, Cd_1, and Cd_5 groups, respectively) and/or extract from *Aronia melanocarpa* L. berries (AE, Cd_1 + AE, and Cd_5 + AE groups) were administered to rats for 3, 10, 17, and 24 months. The presented data are median, minimum, and maximum values for rats in each group (8 animals, except for 7 females in the AE, Cd_1, and Cd_5 groups after 24 months). The statistically significant differences are marked as * $p < 0.05$ and $^{†} p < 0.01$ compared to: a – Control group, b – AE group, c – Cd_1 group, and e – Cd_5 group.

Table S3. The concentrations and contents of calcium (Ca) and magnesium (Mg) in the aliquots of the brain homogenates of female rats.

**Group**	**Experiment Duration**			
	**3 Months**	**10 Months**	**17 Months**	**24 Months**
	**Ca Concentration (µg/g tissue)**			
Control	39.06<br>27.68–45.94	41.52<br>37.25–50.08	56.70<br>42.17–65.78	56.77<br>42.66–72.15
AE	50.57<br>41.32–61.84	42.66<br>36.69–53.51	70.04<br>56.81–80.55	81.35<br>66.11–94.17
Cd_1	47.44<br>38.40–61.48	45.30<br>38.42–51.68	74.60<br>64.69–89.61	92.31 [a‡]<br>82.64–102.1
Cd_1 + AE	54.45 [a†]<br>41.94–63.80	52.73 [a*]<br>45.32–62.92	72.47<br>56.74–91.94	85.31 [a*]<br>66.11–93.12
Cd_5	42.84<br>35.33–49.11	52.19 [a*]<br>49.69–60.44	100.7 [a‡ b*]<br>85.31–116.1	62.86 [c*]<br>55.00–76.46
Cd_5 + AE	53.89 [a†]<br>42.52–60.68	48.07<br>40.96–55.84	76.90<br>61.86–104.9	58.82 [c† d*]<br>41.95–88.89
Effect size (η^2)	0.351	0.322	0.470	0.624
	**Ca Content (µg)**			
Control	63.91<br>49.38–80.35	76.43<br>67.00–96.87	116.6<br>94.85–131.0	105.3<br>89.42–147.1
AE	88.00<br>69.54–112.2	76.90<br>66.74–105.9	143.5<br>112.0–160.4	160.7<br>108.3–194.3
Cd_1	75.55<br>70.93–112.7	85.65<br>73.43–99.96	136.0<br>113.0–197.9	193.0 [a‡]<br>174.5–203.1
Cd_1 + AE	92.88 [a*]<br>66.56–114.5	100.3 [a* b*]<br>87.52–113.8	137.2<br>111.2–184.0	167.3<br>121.6–179.3
Cd_5	71.50<br>59.49–91.27	100.4 [a† b*]<br>92.07–124.5	183.0 [a‡]<br>162.2–216.7	120.6 [c*]<br>106.6–154.4
Cd_5 + AE	103.1 [a‡ e*]<br>89.89–121.2	95.11<br>80.41–100.6	135.0<br>107.1–217.4	108.2 [c†]<br>84.33–195.3
Effect size (η^2)	0.429	0.404	0.376	0.536
	**Mg Concentration (µg/g tissue)**			
Control	154.8<br>148.9–165.0	169.4<br>159.3–180.1	150.7<br>127.2–174.1	141.9<br>124.2–163.6
AE	164.1<br>156.9–171.0	162.3<br>153.6–177.2	161.9<br>151.5–178.3	143.9<br>130.0–174.3
Cd_1	159.0<br>147.7–180.8	162.5<br>155.4–179.5	157.0<br>139.4–166.9	145.0<br>119.9–165.4
Cd_1 + AE	179.6 [a*]<br>157.4–198.6	175.8<br>164.8–189.4	159.0<br>146.8–166.3	148.4<br>128.1–185.8
Cd_5	157.7<br>149.2–175.2	168.4<br>150.8–178.2	170.1 [a*]<br>153.4–186.5	151.8<br>138.3–169.9
Cd_5 + AE	154.4 [d*]<br>140.0–172.1	168.7<br>156.2–172.4	161.0<br>151.2–198.7	138.5<br>130.8–166.5
Effect size (η^2)	0.117	-	0.01	-
	**Mg Content (µg)**			
Control	256.9<br>215.1–320.4	321.7<br>297.9–348.4	310.4<br>257.0–333.8	293.9<br>229.8–320.8

AE	292.7<br>270.3–318.1	304.8<br>274.3–340.0	329.8<br>272.6–368.7	284.0<br>210.9–342.4
Cd_1	283.2<br>269.3–332.8	326.6<br>278.1–332.3	292.3<br>257.5–353.3	294.0<br>250.1–355.5
Cd_1 + AE	304.9 [a*]<br>252.8–373.4	336.4<br>311.4–370.4	314.2<br>276.7–327.9	284.3<br>240.8–386.7
Cd_5	282.2<br>242.1–320.1	331.1<br>285.7–360.7	324.1<br>236.7–381.4	306.3<br>275.7–343.2
Cd_5 + AE	300.2<br>271.5–334.2	314.8<br>303.3–336.9	322.6<br>280.8–386.1	294.3<br>252.7–367.2
Effect size (η^2)	0.162	-	-	-

Cadmium (Cd) (0, 1, or 5 mg Cd/kg, respectively, for the Control, Cd_1, and Cd_5 groups) and/or extract from *Aronia melanocarpa* L. berries (AE, Cd_1 + AE, and Cd_5 + AE groups) were administered to rats for 3, 10, 17, and 24 months. The presented data are median, minimum and maximum values for rats in each group (8 animals, except for 7 females in the AE, Cd_1, and Cd_5 groups after 24 months). The statistically significant differences are marked as * $p < 0.05$, and † $p < 0.01$ compared to: a – Control group, b – AE group, c – Cd_1 group, d – Cd_1 + AE group, and e – Cd_5 group.

Table S4. The concentrations of calmodulin (CAM), phospholipase A2 (PLA2), and nitric oxide synthase 1 (NOS1) in the aliquots of the brain homogenates of female rats.

Group	Experiment Duration			
	**3 Months**	**10 Months**	**17 Months**	**24 Months**
	**CAM (ng/mg protein)**			
Control	1.214<br>1.133–3.439	1.016<br>0.218–2.333	0.875<br>0.700–1.034	0.617<br>0.511–0.798
AE	1.594<br>0.576–4.493	1.040<br>0.397–1.502	0.881<br>0.762–1.524	0.703<br>0.527–1.978
Cd$_1$	1.233<br>0.365–1.760	1.216<br>0.798–2.434	2.200<br>1.650–4.027	2.194 ^{a*}<br>1.879–3.639
Cd$_1$ + AE	0.650 ^{b*}<br>0.356–1.415	1.144<br>0.398–1.580	0.668 ^{c†}<br>0.581–0.945	0.623 ^{c*}<br>0.481–1.790
Cd$_5$	1.173<br>0.716–4.016	1.262<br>0.660–1.808	2.393 ^{d‡}<br>1.753–2.684	2.292 $^{a\dagger\ d*}$<br>2.162–2.458
Cd$_5$ + AE	0.685 ^{b*}<br>0.485–1.126	0.668<br>0.377–1.372	0.642 $^{c\ddagger\ e\ddagger}$<br>0.457–0.815	0.612 $^{c\dagger\ e\ddagger}$<br>0.516–0.670
Effect size (η^2)	0.327	-	0.818	0.638
	**PLA2 (ng/mg protein)**			
Control	1.969<br>1.617–2.705	1.784<br>0.790–3.089	3.398<br>2.557–4.336	3.066<br>2.541–4.067
AE	1.106<br>0.675–3.647	1.530<br>0.666–3.502	1.425<br>0.981–2.538	2.720<br>2.250–3.858
Cd$_1$	0.628<br>0.348–2.146	0.965<br>0.564–1.258	0.609 ^{a‡}<br>0.426–1.024	0.594 $^{a\dagger\ b*}$<br>0.419–0.918
Cd$_1$ + AE	1.840<br>1.097–4.356	1.683 ^{c*}<br>1.293–3.683	2.270<br>1.311–3.153	2.071<br>1.277–3.323
Cd$_5$	1.995<br>1.413–4.759	0.689 $^{a\dagger\ b*\ d\dagger}$<br>0.192–1.084	0.558 ^{a‡}<br>0.527–0.813	0.408 $^{a\ddagger\ b\ddagger}$<br>0.354–0.535
Cd$_5$ + AE	2.211 ^{c*}<br>0.760–4.389	1.629 ^{e*}<br>0.948–2.877	2.820 $^{c\dagger\ e\ddagger}$<br>2.332–3.675	1.828 ^{e*}<br>1.540–4.230
Effect size (η^2)	0.276	0.477	0.838	0.755
	**NOS1 (ng/mg protein)**			
Control	0.573<br>0.352–1.089	0.304<br>0.203–0.411	0.364<br>0.259–0.646	0.285<br>0.213–0.522
AE	0.458<br>0.230–1.026	0.227<br>0.160–0.327	0.304<br>0.220–0.563	0.341<br>0.227–0.501
Cd$_1$	0.276 ^{a†}<br>0.220–0.337	0.228<br>0.192–0.282	0.273<br>0.191–0.363	1.638 $^{a\dagger\ b*}$<br>0.986–2.607
Cd$_1$ + AE	0.443<br>0.269–0.854	0.221<br>0.162–0.305	0.355<br>0.190–0.588	0.490 ^{c*}<br>0.199–1.404
Cd$_5$	0.511 ^{c‡}<br>0.390–0.665	0.255<br>0.136–0.379	0.294<br>0.199–0.360	1.122 ^{a*}<br>0.821–1.557
Cd$_5$ + AE	0.349<br>0.284–0.428	0.325<br>0.193–0.528	0.276<br>0.210–0.347	0.265 $^{c\dagger\ e*}$<br>0.172–0.416
Effect size (η^2)	0.403	-	-	0.575

Cadmium (Cd) (0, 1 or 5 mg Cd/kg, respectively, for the Control, Cd$_1$, and Cd$_5$ groups) and/or extract from *Aronia melanocarpa* L. berries (AE, Cd$_1$ + AE, and Cd$_5$ + AE groups) were administered to rats for 3, 10, 17, and 24 months. The presented data are median, minimum and maximum values for rats in each group (8 animals, except for 7 females in the AE, Cd$_1$, and Cd$_5$ groups after 24 months). The statistically significant differences are marked as * $p < 0.05$, $^{\dagger}p < 0.01$, and $^{\ddagger}p < 0.001$ compared to: a – Control group, b – AE group, c – Cd$_1$ group, d – Cd$_1$ + AE group, and e – Cd$_5$ group.

Table S5. The concentrations of nuclear factor erythroid 2-related factor 2 (Nrf2) and Kelch-like ECH-associated protein 1 (KEAP1) in the aliquots of the brain homogenates of female rats.

Group	Experiment Duration			
	**3 Months**	**10 Months**	**17 Months**	**24 Months**
**Nrf2 (pg/mg protein)**				
Control	277.8<br>145.1–654.3	173.3<br>126.5–225.1	223.5<br>147.3–479.6	148.7<br>118.9–217.6
AE	196.2<br>127.2–415.2	115.6<br>91.99–163.3	203.7<br>143.2–283.8	77.76<br>58.19–94.65
Cd_1	122.4 ^{a‡}<br>95.35–152.0	102.1 ^{a†}<br>92.11–115.0	71.74 ^{a*} ^{b*}<br>40.96–92.24	72.01 ^{a*}<br>47.72–97.83
Cd_1 + AE	176.4<br>117.9–420.1	114.0<br>86.20–144.1	132.8<br>44.57–230.3	69.07 ^{a†}<br>53.05–100.3
Cd_5	162.1<br>130.6–215.3	114.8 ^{a*}<br>78.53–167.4	67.98 $^{a\dagger\, b\dagger}$<br>30.77–84.33	69.71 ^{a*}<br>54.15–85.38
Cd_5 + AE	177.9 ^{c*}<br>152.4–212.2	135.9<br>113.2–153.9	51.76 $^{a\ddagger\, b\ddagger}$<br>38.17–92.86	67.79 ^{a†}<br>40.82–76.36
Effect size (η^2)	0.383	0.389	0.723	0.407
**KEAP1 (ng/mg protein)**				
Control	0.898<br>0.611–1.617	0.772<br>0.468–1.126	0.656<br>0.428–0.940	0.525<br>0.297–0.708
AE	0.590<br>0.294–1.404	0.634<br>0.443–0.924	0.729<br>0.215–2.993	0.235<br>0.035–0.444
Cd_1	0.332 ^{a‡}<br>0.277–0.390	0.410 ^{a*}<br>0.287–0.539	0.319<br>0.094–0.665	0.184 ^{a†}<br>0.070–0.278
Cd_1 + AE	0.450 ^{a†}<br>0.289–0.863	0.373 $^{a\dagger\, b*}$<br>0.231–0.642	0.288 ^{a*}<br>0.108–0.457	0.209 ^{a*}<br>0.086–0.409
Cd_5	0.489<br>0.373–0.686	0.386 ^{a†}<br>0.292–0.574	0.290 ^{a*}<br>0.095–0.558	0.170 ^{a†}<br>0.068–0.317
Cd_5 + AE	0.507<br>0.368–0.675	0.417 ^{a*}<br>0.338–0.607	0.453<br>0.220–0.835	0.232<br>0.146–0.292
Effect size (η^2)	0.476	0.495	0.248	0.335

Cadmium (Cd) (0, 1 or 5 mg Cd/kg, respectively, for the Control, Cd_1, and Cd_5 groups) and/or extract from *Aronia melanocarpa* L. berries (AE, Cd_1 + AE, and Cd_5 + AE groups) were administered to rats for 3, 10, 17, and 24 months. The presented data are median, minimum, and maximum values for rats in each group (8 animals, except for 7 females in the AE, Cd_1, and Cd_5 groups after 24 months). The statistically significant differences are marked as * $p < 0.05$, $^{\dagger} p < 0.01$, and $^{\ddagger} p < 0.001$ compared to: a – Control group, b – AE group, and c – Cd_1 group.

Table S6. The concentrations of metalloproteinases (MMPs): MMP-2, MMP-3, and MMP-9 in the aliquots of the brain homogenates of female rats.

Group	Experiment Duration			
	**3 Months**	**10 Months**	**17 Months**	**24 Months**
**MMP-2 (ng/mg protein)**				
Control	6.483<br>2.981–8.618	6.170<br>5.397–8.940	6.452<br>4.480–9.277	6.851<br>5.845–8.843
AE	9.006<br>6.227–20.92	4.918<br>2.505–8.303	6.487<br>4.844–8.039	6.084<br>4.978–11.74
Cd_1	5.870<br>4.895–7.600	6.227<br>4.998–8.462	6.233<br>4.198–9.497	4.450 ^{a†}<br>3.701–5.228
Cd_1 + AE	5.799<br>2.919–16.67	5.894<br>4.575–7.702	6.188<br>4.855–9.021	6.224 ^{c*}<br>5.346–8.528
Cd_5	9.105<br>5.470–14.96	5.712<br>5.270–7.670	5.217<br>4.661–6.189	5.282 ^{a*}<br>3.003–6.018
Cd_5 + AE	8.302<br>7.457–10.58	8.787 $^{b\ddagger\ d*\ e*}$<br>6.948–9.388	6.751 ^{e*}<br>5.927–8.900	6.323 ^{c*}<br>5.044–7.338
Effect size (η^2)	-	0.341	0.128	0.451
**MMP-3 (ng/mg protein)**				
Control	1.458<br>0.403–2.637	1.217<br>0.992–1.367	0.432<br>0.143–0.837	1.235<br>1.062–2.487
AE	1.508<br>0.962–5.997	1.427<br>0.705–3.041	0.414<br>0.154–0.661	1.236<br>1.076–4.384
Cd_1	1.075<br>0.586–1.537	0.792<br>0.272–1.739	1.069<br>0.512–1.447	1.212<br>0.875–1.431
Cd_1 + AE	1.230<br>0.143–4.207	0.828<br>0.130–1.617	1.054<br>0.575–1.573	1.109<br>0.520–1.715
Cd_5	1.184<br>0.160–3.145	1.086<br>0.626–1.651	0.974<br>0.555–1.393	1.022<br>0.513–1.503
Cd_5 + AE	1.918<br>0.823–2.127	1.747 $^{c\dagger\ d\dagger\ e*}$<br>1.302–2.121	1.652 $^{a\ddagger\ b\ddagger}$<br>1.029–3.647	0.676 $^{a*\ b*}$<br>0.400–1.064
Effect size (η^2)	-	0.377	0.546	0.289
**MMP-9 (ng/mg protein)**				
Control	27.92<br>23.56–38.93	24.32<br>19.61–36.82	25.11<br>23.02–48.03	25.46<br>20.40–28.74
AE	34.50<br>24.59–42.02	21.22<br>18.21–26.73	26.93<br>22.21–50.51	23.24<br>9.953–41.95
Cd_1	50.51 ^{a*}<br>37.89 – 57.61	23.20<br>20.48–35.01	24.62<br>19.98–38.32	36.30<br>31.48–68.38
Cd_1 + AE	25.26 ^{c*}<br>21.54–51.89	24.01<br>18.09–33.43	19.98<br>16.63–29.19	18.88 ^{c†}<br>15.18–29.54
Cd_5	44.35<br>32.75–70.12	53.54 $^{a*\ b\ddagger\ c*\ d\dagger}$<br>43.36–70.16	49.05 ^{d‡}<br>43.86–56.59	37.18 ^{d*}<br>21.98–43.59
Cd_5 + AE	24.93 $^{c\dagger\ e\dagger}$<br>11.79–33.61	24.44 ^{e*}<br>21.49–31.88	18.97 $^{b*\ e\ddagger}$<br>16.05–21.77	19.54 $^{c\ddagger\ e\dagger}$<br>18.92–20.74
Effect size (η^2)	0.500	0.388	0.683	0.552

Cadmium (Cd) (0, 1 or 5 mg Cd/kg, respectively, for the Control, Cd_1, and Cd_5 groups) and/or extract from *Aronia melanocarpa* L. berries (AE, Cd_1 + AE, and Cd_5 + AE groups) were administered to rats for 3, 10, 17, and 24 months. The presented data are median, minimum, and maximum values for rats in each group (8 animals, except for 7 females in the AE, Cd_1, and Cd_5 groups after 24 months). The statistically significant differences are marked as * $p < 0.05$, † $p < 0.01$, and ‡ $p < 0.001$ compared to: a – Control group, b – AE group, c – Cd_1 group, d – Cd_1 + AE group, and e – Cd_5 group.

Table S7. The concentrations of tissue inhibitors of metalloproteinases (TIMPs): TIMP-2, TIMP-3, and TIMP-4 in the aliquots of the brain homogenates of female rats.

**Group**	**Experiment Duration**			
	**3 Months**	**10 Months**	**17 Months**	**24 Months**
		**TIMP-2 (ng/mg protein)**		
Control	16.70<br>13.95–32.04	14.53<br>12.70–25.76	15.82<br>12.37–23.50	15.75<br>13.76–22.50
AE	20.38<br>13.54–54.44	12.42<br>6.972–23.15	16.39<br>13.52–26.30	19.61<br>15.95–27.25
Cd$_1$	16.83<br>13.36–19.90	14.34<br>11.92–20.25	15.24<br>11.67–26.24	17.02<br>12.68–28.79
Cd$_1$ + AE	16.85<br>13.48–37.26	13.06<br>10.73–19.99	16.15<br>12.87–22.62	17.63<br>10.56–31.15
Cd$_5$	21.92<br>11.12–35.08	15.43<br>13.05–24.76	15.26<br>11.39–19.34	12.05 ^{b*}<br>8.020–14.09
Cd$_5$ + AE	19.54<br>15.38–27.54	16.83<br>11.95–21.68	15.56<br>13.13–18.53	28.75 $^{a*\ e‡}$<br>21.78–34.08
Effect size (η^2)	-	-	-	0.566
		**TIMP-3 (ng/mg protein)**		
Control	2.720<br>1.799–3.064	5.419<br>3.928–7.216	5.773<br>3.892–7.086	5.872<br>4.537–6.994
AE	2.686<br>1.069–3.568	4.957<br>3.160–10.30	5.827<br>5.061–9.399	6.281<br>6.083–12.27
Cd$_1$	3.401<br>2.859–4.427	3.417 ^{a*}<br>1.374–5.101	6.256<br>5.077–10.02	5.982<br>5.225–9.510
Cd$_1$ + AE	3.339<br>1.465–3.594	4.236<br>2.414–5.997	5.237<br>4.792–9.517	5.434<br>4.094–8.513
Cd$_5$	2.717<br>1.861–3.445	4.518<br>2.992–5.978	6.052<br>4.082–7.319	6.389<br>5.984–8.026
Cd$_5$ + AE	2.825<br>2.134–2.931	4.403<br>3.623–6.821	5.748<br>4.982–6.557	1.644 $^{b‡\ c*\ e‡}$<br>0.558–2.958
Effect size (η^2)	-	0.131	-	0.531
		**TIMP-4 (ng/mg protein)**		
Control	10.66<br>6.451–19.71	5.243<br>4.641–8.861	7.438<br>5.550–9.473	6.388<br>6.187–7.706
AE	8.160<br>6.082–20.50	5.238<br>4.466–7.061	6.343<br>5.180–11.39	6.585<br>4.236–13.51
Cd$_1$	7.139<br>4.901–8.002	6.113<br>5.116–8.084	6.588<br>5.481–9.962	7.059<br>4.447–11.42
Cd$_1$ + AE	7.343<br>6.143–15.97	5.467<br>2.818–8.412	11.65 $^{b†\ c*}$<br>9.554–16.99	10.86 ^{a*}<br>9.070–16.43
Cd$_5$	5.693 ^{a‡}<br>5.100–6.551	4.983<br>3.340–7.025	6.483 ^{d†}<br>5.198–7.750	6.565<br>3.431–10.63
Cd$_5$ + AE	9.133 ^{e†}<br>7.153–11.78	10.31 $^{b‡\ d*\ e†}$<br>7.142–14.10	11.42 $^{b†\ c*\ e†}$<br>9.262–13.50	12.79 $^{a†\ b*\ c*\ e*}$<br>11.06–14.15
Effect size (η^2)	0.411	0.354	0.611	0.545

Cadmium (Cd) (0, 1 or 5 mg Cd/kg, respectively, for the Control, Cd$_1$, and Cd$_5$ groups) and/or extract from *Aronia melanocarpa* L. berries (AE, Cd$_1$ + AE, and Cd$_5$ + AE groups) were administered to rats for 3, 10, 17, and 24 months. The presented data are median, minimum, and maximum values for rats in each group (8 animals, except for 7 females in the AE, Cd$_1$, and Cd$_5$ groups after 24 months). The statistically significant differences are marked as * $p < 0.05$, † $p < 0.01$, and ‡ $p < 0.001$ compared to: a – Control group, b – AE group, c – Cd$_1$ group, d – Cd$_1$ + AE group, and e – Cd$_5$ group.
